# Metabolic efficiency reshapes the seminal relationship between pathogen growth rate and virulence

**DOI:** 10.1111/ele.14218

**Published:** 2023-04-13

**Authors:** Richard J. Lindsay, Philippa J. Holder, Nicholas J. Talbot, Ivana Gudelj

**Affiliations:** ^1^ Biosciences and Living Systems Institute University of Exeter Exeter UK; ^2^ The Sainsbury Laboratory University of East Anglia, Norwich Research Park Norwich UK

**Keywords:** growth rate, metabolic efficiency, microbial ecology, plant pathogens, synthetic ecology, trade‐offs, virulence

## Abstract

A cornerstone of classical virulence evolution theories is the assumption that pathogen growth rate is positively correlated with virulence, the amount of damage pathogens inflict on their hosts. Such theories are key for incorporating evolutionary principles into sustainable disease management strategies. Yet, empirical evidence raises doubts over this central assumption underpinning classical theories, thus undermining their generality and predictive power. In this paper, we identify a key component missing from current theories which redefines the growth–virulence relationship in a way that is consistent with data. By modifying the activity of a single metabolic gene, we engineered strains of *Magnaporthe oryzae* with different nutrient acquisition and growth rates. We conducted *in planta* infection studies and uncovered an unexpected non‐monotonic relationship between growth rate and virulence that is jointly shaped by how growth rate and metabolic efficiency interact. This novel mechanistic framework paves the way for a much‐needed new suite of virulence evolution theories.

## Graphical abstract

A cornerstone assumption underpinning existing virulence evolution theories is that pathogen growth rate positively correlates with virulence. Yet there is growing empirical evidence that this is far from universal. In this paper, we identify a key component missing from current theories which enables us to reformulate the growth–virulence relationship in a way that is, for the first time, consistent with data.

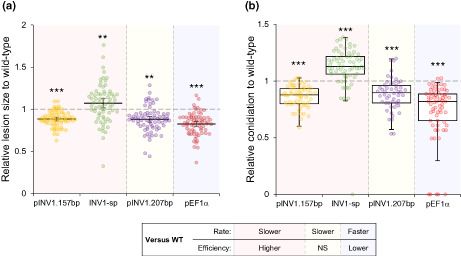

## PEER REVIEW

The peer review history for this article is available at https://www.webofscience.com/api/gateway/wos/peer‐review/10.1111/ele.14218.

## INTRODUCTION

Why do pathogens harm their host on which they rely? Why are some pathogens highly damaging while others are benign? These questions have been at the heart of the evolutionary ecology field for over three decades, and have featured prominently in theoretical literature. This vast theoretical research has taught us that the reduction in host fitness caused by a pathogen (termed virulence) is influenced by evolutionary trade‐offs at both the within‐ and between‐host scale (reviewed in Cressler et al., 2016). Within the host, pathogens are thought to inevitably cause damage when exploiting host‐derived nutrients to fuel their own growth and replication. While numerous system‐specific features of the pathogen–host interaction contribute towards virulence (e.g. Blanquart et al., 2016), a key theoretical assumption is that resource consumption, and the resulting growth rate, positively correlates with virulence (Anderson & May, 1982; Bremermann & Pickering, 1983; Choisy & de Roode, 2010; Frank, 1996; Lenski & May, 1994; Levin & Bull, 1994; Nowak & May, 1994; van Baalen & Sabelis, 1995). This forms the basis for the majority of our understanding regarding the evolution of virulence (Cressler et al., 2016). In the context of co‐infection, faster growing and more virulent pathogens are expected to be favoured (Alizon et al., 2013). Yet despite some empirical support (e.g. Ben‐Ami et al., 2008; De Roode et al., 2005; De Roode et al., 2008; Herre, 1993; Kerr et al., 2006; Zhan et al., 2016), there is often limited evidence that growth rate and virulence are positively correlated (e.g. Duxbury et al., 2020; Mikonranta et al., 2012; Pagán et al., 2007; Tardy et al., 2019; Zhan et al., 2002). Sometimes slower growing pathogens are even found to be more virulent than faster growers (e.g. Gower & Webster, 2005; Leggett et al., 2017; Little et al., 2008; Meyer et al., 2010), putting into question the generality of current theories and their predictive capability.

We thus urgently need a mechanistic understanding of the relationship between pathogen growth rate and virulence. Most existing theories reason that faster growing pathogens are more virulent because they consume host resources quicker than slower growers (Anderson & May, 1982; Bremermann & Pickering, 1983; Choisy & de Roode, 2010; Frank, 1996; Lenski & May, 1994; Levin & Bull, 1994; Nowak & May, 1994; van Baalen & Sabelis, 1995). In contrast, auxiliary pathogen traits could break down the positive relationship between growth rate and virulence. For example, slower growing pathogens might be more virulent because they can divert more energy towards other disease‐specific processes, such as the production of virulence factors (Cui et al., 2019; Meyer et al., 2010; Peyraud et al., 2016; Sturm et al., 2011). Similarly, virulence is influenced by other system‐specific features of the infection process (Leggett et al., 2017), including interactions with the host immune system (Frank & Schmid‐Hempel, 2008; Tardy et al., 2019), and the production of effector proteins (Yan et al., 2023). Here, we argue that this is not the complete picture.

We show that the positive relationship between growth rate and virulence could also break down due to a ubiquitous metabolic feature of growth, rather than due to system‐specific features of the disease process. We hypothesised that a key component of microbial metabolism missing from current theories could provide a unifying explanation for why some studies observe a positive while others observe a negative relationship between growth rate and virulence. Namely, when metabolising nutrients for growth, organisms frequently experience a rate‐efficiency trade‐off (RETO), which is considered to be a thermodynamic necessity (Pfeiffer et al., 2001) and has been observed across microbial taxa (Beardmore et al., 2011; Nev et al., 2021). This trade‐off means that ATP and, subsequently, biomass can be produced either more rapidly (growth per unit of time) or more efficiently (growth per unit of resource). Which strategy is implemented tends to depend on resource uptake rates, with fast but inefficient metabolism used when resource levels are high (Beardmore et al., 2011; Lipson, 2015; Postma et al., 1989). Therefore, slower growing organisms can increase their yield of biomass production because they metabolise substrates more efficiently. Certain conditions that pathogens often experience, including low resource concentrations, and spatial and temporal heterogeneity of resources, can increase the fitness of efficient metabolism. Since the RETO can influence an organism's competitive ability and virulence (Lindsay et al., 2016; MacLean & Gudelj, 2006; Pfeiffer et al., 2001), we hypothesise that it will also impact the growth rate–virulence relationship and hence virulence evolution (Wale, 2022).

A common shortfall of past studies on how growth impacts virulence is that they routinely compare pathogens with undefined genetic differences (Alizon et al., 2013; Buckling & Brockhurst, 2008). As a result, the mechanisms behind observed variations cannot be precisely attributed to differences in growth properties. This is due to confounding effects, such as differences in host responses to genetically diverse pathogens (e.g. Taylor et al., 1998), or unknown interactions between pathogen strains (e.g. Davies et al., 2002). Moreover, measures of virulence can vary between host genotypes when pathogen genetic differences are undefined and relate to diverse aspects of infection (Young et al., 2018; Zhan et al., 2002).

To overcome this, we engineered strains of the rice blast fungus, *Magnaporthe oryzae*, with different growth properties but in an otherwise isogenic background. *M. oryzae* causes blast disease wherever rice is cultivated. It is a significant threat to global rice production and a key model system for studying plant‐pathogen interactions (Ebbole, 2007; Fernandez & Orth, 2018; Martin‐Urdiroz et al., 2016). In addition, while metabolising sucrose, the product of photosynthesis and the primary storage sugar within plants, *M. oryzae* is known to be constrained by the RETO (Lindsay et al., 2016), like many other microbial species. Therefore, the higher the resource concentration, the faster the growth rate and the lower the growth efficiency.

The key to our synthetic pathogen library is a single gene (*INV1*) whose manipulation alters resource supply rates to the pathogen and therefore its growth rate. During infection of rice, *M. oryzae* invades plant tissue and acquires nutrients, including sucrose, to fuel growth and conidia production for transmission (Fernandez & Orth, 2018; Martin‐Urdiroz et al., 2016). Like other plant‐pathogenic fungi (Parrent et al., 2009), it produces an invertase enzyme, encoded by *INV1*, to extracellularly hydrolyse sucrose into its constituent hexoses, glucose and fructose (Lindsay et al., 2016). *M. oryzae* dynamically regulates carbon metabolism to differing nutritional environments during infection. It prudently controls metabolic gene expression between the nutrient poor leaf surface, to the initially nutrient rich leaf apoplast, followed by de‐repression of genes involved in the metabolism of polymeric carbon sources after readily available sugars have been consumed (Fernandez & Orth, 2018; Foster et al., 2016).

In this study, we modified INV1 activity in *M. oryzae* strains to manipulate resource consumption rate, thus enabling us to examine the relationship between growth rate and virulence in a controlled manner. We found this relationship was non‐monotonic and jointly shaped by the interaction between pathogen growth efficiency (quantified as the yield of reproductive conidia per resource) and growth rate (measured as biomass per unit of time), with the latter found to be representative of growth rate *in planta*. Subsequently, we explored the consequences of this finding for virulence evolution. In agreement with classical studies (Frank, 1996; Levin & Bull, 1994; Nowak & May, 1994), we found that faster growing strains have a competitive advantage in co‐inoculation infection studies. However, the RETO meant that faster growing pathogens could have lower metabolic efficiency than slower growing ones, which could in turn make them less virulent.

Our controlled synthetic experimental system contributes new mechanistic understanding of the relationship between growth rate and virulence. Importantly, it provides a framework for interpreting the impact of pathogen growth rate on virulence, fundamental for understanding the driving forces of virulence evolution.

## RESULTS

### The rate‐efficiency trade‐off in wild type *M. oryzae*

**FIGURE 1:**
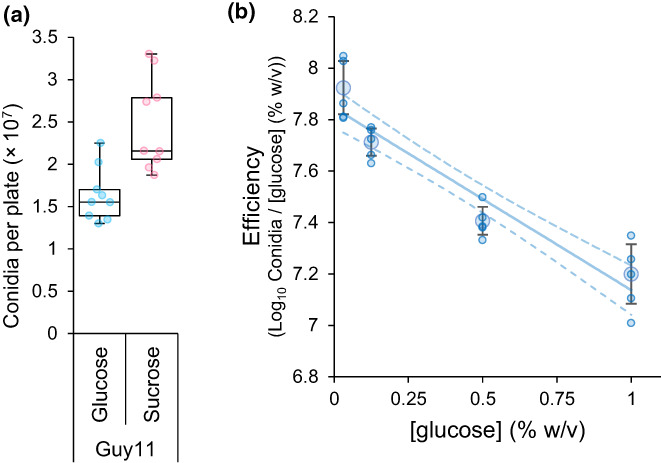
(a) The wt (Guy11) metabolised 1% glucose MM less efficiently than 1% sucrose MM, in terms of the number of conidia produced per plate (two‐sample, two‐sided *t‐*test: *p* < 1.17 × 10^−3^, *t* = 3.94, *n* = 9). (b) Efficiency decreases with increasing glucose concentration (measured as Log_10_(conidia yield) of wild‐type Guy11 per unit of resource (glucose %) after 12 days on glucose‐supplemented MM ‐ linear model: *F*_(1, 18)_ = 102.8, *p* < 7.21 × 10^−9^, Adj. *R* ^2^ = 0.8428, Line shows model ± 95% CI (dashed lines). Fligner–Killeen: *χ* ^2^ = 3.0845, *p* = 0.3788. Shapiro–Wilk: *W* = 0.91374, *p* = 0.07514). Large markers show mean ± 95% CI, small markers show all replicates, *n* = 5 per concentration.

*In vitro* growth of the wild‐type, Guy11, is constrained by the RETO. The extra catabolic step during sucrose hydrolysis can slow resource consumption when metabolising sucrose compared to glucose (Lindsay et al., 2019). Indeed, we observed that Guy11 metabolised glucose less efficiently than sucrose, in terms of number of conidia produced per resource (Figure 1a).

Further evidence of the RETO comes from when the efficiency of Guy11 increased when glucose concentrations decreased from 1% to 0.03125% (Figure 1b). Since resources are taken up quicker at higher than lower concentrations (Gudelj et al., 2007), the RETO in microorganisms is routinely demonstrated by observing changes in efficiency wile varying resource concentrations (Lindsay et al., 2018; Meyer et al., 2015; Nev et al., 2021; Postma et al., 1989). To investigate the effect of the RETO on pathogen virulence *in vivo*, where resource concentrations cannot be readily controlled, we instead sought to generate a range of *M. oryzae* strains that differ in sucrose metabolism rate and consequently efficiency.

### Generating a library of *M. oryzae* strains with modified invertase expression

**FIGURE 2:**
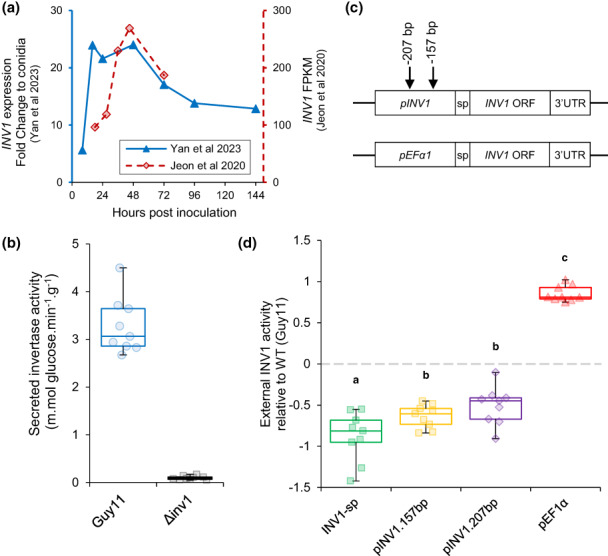
*INV1* expression and alteration by 5′ UTR modification. (a) *INV1* is expressed by wild‐type *M. oryzae* throughout the rice infection process. Data shows fold change in expression to conidia from Yan et al. (2023) and fragments per kilobase of exon model per million reads mapped from Jeon et al. (2020). (b) The invertase null‐mutant (Δ*inv1*) is deficient in sucrose hydrolysis. (c) A library of strains were engineered with altered *INV1* expression. Modifications were either 5′ truncations of the native promoter ( *pINV1* ) to 157 bp or 207 bp upstream of the start codon, deletion of the secretion signal peptide (sp) sequence (INV1‐sp), or swapping the native promoter with the *EF1α* promoter. Not to scale. (d) The extracellular invertase activity of the resulting strains was assessed by enzymatic assay of live mycelia. INV1‐sp, pINV1.157bp and pINV1.207bp had lower activity than the wild‐type, whereas pEF1α had increased activity (Two‐sample two‐sided *t*‐test: *p* < 0.0001). Box plots show median, 25/75 percentiles and min/max. Data for each strain has been normalised against the wild‐type activity (${\mathrm{Log}}_{10}({\text{mutant}}_{i}\;$/$\;\bar{\mathrm{wt}})$), measured concurrently for each experimental repeat (equal activity = 0, indicated by dashed line). Different letters indicate significant differences between relative activity (Linear model: relative activity with respect to Strain). See Figure S1 for full dataset and Table S1 for full data analysis. Markers show all replicates, *n* = 9.

We developed a collection of otherwise isogenic pathogen strains with different growth rates. In the wild‐type, *INV1* is expressed throughout the biotrophic and necrotrophic phases of infection (Figure 2a) (Jeon et al., 2020; Yan et al., 2023). Sucrose is a key carbon source for *M. oryzae* during infection, as demonstrated by deletion of *INV1*, which renders *M. oryzae* unable to metabolise sucrose (Figure 2b) and inhibits successful infection (Lindsay et al., 2016). Thus, to manipulate growth rate we modified the pathogen's sucrose hydrolysis rate and resource consumption by altering *INV1* expression levels or subcellular localisation. We used the wild‐type Guy11 as a baseline of *INV1* expression and growth properties, from which modifications generated an additional four strains.

In each strain, *INV1* expression was modified using one of three approaches: promoter swapping, 5′ promoter truncation or secretion signal peptide deletion (Figure 2c). Three of the resulting strains (pINV1.157bp, pINV1.207bp and INV1‐sp) had lower *INV1* activity than the wild‐type. The strains pINV1.157bp and pINV1.207bp had 5′ promoter truncations that can alter transcriptions levels (Soanes et al., 2002) and INV1‐sp had a secretion signal peptide deletion (Figure S2), which has previously been shown to reduce the rate of sucrose metabolism and growth (Lindsay et al., 2019). Conversely, strain pEF1α had its native promoter swapped with that of the elongation factor 1‐alpha gene (*EF1α*), which resulted in higher *INV1* activity than the wild‐type (Figure 2d, Figure S1 – see methods for more details).

### Altering *INV1‐*mediated sucrose metabolism alters *M. oryzae* growth properties

The growth properties of the strains with altered *INV1* expression were assessed *in vitro*, as commonly done in virulence studies (Leggett et al., 2017; Meyer et al., 2010; Peyraud et al., 2016; Sturm et al., 2011; Zhan et al., 2016). Different growth parameters during the *M. oryzae* life cycle were quantified on 1% sucrose minimal media (MM), mirroring concentrations experienced during infection (Dallagnol et al., 2013; Saito & Yoshida, 2011). Specifically, growth rate was measured by biomass formation in liquid media while resources were abundant to ensure that growth had not decelerated or biomass senesced. Growth efficiency was measured by conidia production per resource on agar‐supplemented media, allowing resources to be exhausted, which subsequently induced conidiation (See Methods S1 for further measurement details and justification).

**FIGURE 3:**
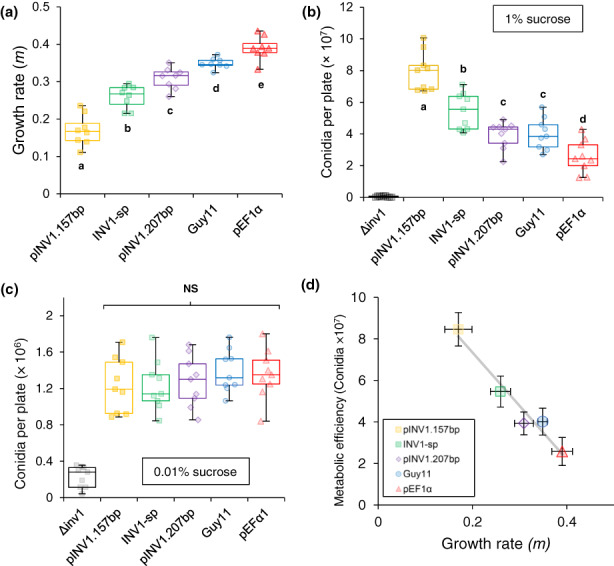
Growth properties of strains with altered *INV1* expression. (a) Strains have different growth rates in liquid 1% sucrose MM (linear model: *p* < 2.54 × 10^−15^, *F*_(4,35)_ = 61.29, Adj. *R* ^2^ = 0.861. *n* = 8). See Figure S3 for time‐series plot. (b) Metabolic efficiency (measured as conidia production on 1% sucrose MM + agar) varied between strains (linear model: *p* < 3.28 × 10^−12^, *F*_(4,40)_ = 33.14, Adj. *R* ^2^ = 0.745, *n* = 9). (c) This difference in metabolic efficiency was lost when equivalent growth assays were conducted on 0.01% sucrose MM + agar, where nutrient consumption is slow for all strains (linear model: *p* = 0.647, *F*_(4,40)_ = 0.625, *n* = 9). See Table S2 for full data analysis. (a–c) Markers show all replicates. Box plots show median, 25/75 percentiles and min/max. Labels indicate sucrose concentration (w/v). (d) A between‐strain RETO was evident in the strain library (linear model on means: *p* < 2.85 × 10^−3^, *F*_(1,3)_ = 81.89, Adj. *R* ^2^ = 0.953, replicates shown in (a, b). Markers show mean ± 95% CI.

We found that on sucrose media the engineered strains differed in their growth rates (Figure 3a) and efficiencies (Figure 3b). We also further verified that this outcome was caused by the RETO rather than, for example, unidentified pleiotropy, off‐target effects, differing metabolic burden of *INV1* expression or disparity in maintenance energy requirements (Lipson, 2015). This was achieved by repeating the metabolic efficiency tests but with a low sucrose concentration (0.01%) where the RETO is weak or absent (Lindsay et al., 2016; Postma et al., 1989). Under these conditions, all strains with modified *INV1* expression metabolised resources slowly and with equivalent efficiency (Figure 3c). Thus, we found that modifying *INV1* expression alters growth rate and metabolic efficiency, demonstrating a between‐strain rate‐efficiency trade‐off (Figure 3d). This relationship between, growth rate and metabolic efficiency in engineered strains is analogous to when the wild‐type is provided with different resource types and concentrations *in vitro* (Figure 1) and has also been observed in diverse microbial taxa, including human pathogens (Nev et al., 2021).

### Growth properties influence disease virulence

**FIGURE 4:**
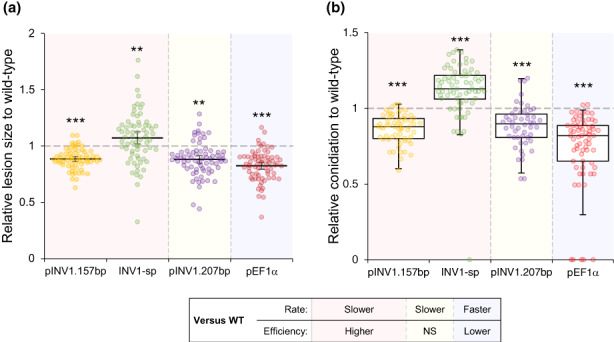
Infection assay of *M. oryzae* mutants with modified growth properties. Disease virulence and pathogen conidiation were measured by leaf drop inoculation of rice cultivar CO‐39. Virulence and conidiation of each strain were normalised against mean lesion area (due to approximately normally distributed data) or median conidiation (due to non‐normal distribution) of the wild‐type (Guy11 ‐ WT) to aid visualisation, which were measured concurrently with each mutant. (a) Lesion area was quantified by image analysis of disease lesions after 7 days. The WT was more virulent than pINV1.157bp, pINV1.207bp and pEF1α, whereas INV1‐sp was more virulent than WT (two‐sample, two‐sided *t*‐test (each strain vs. WT): **p < 0.01, ***p < 0.001). Mean ± 95% CI. (b) Pathogen conidiation was quantified from disease lesions. WT produced more conidia per lesion than pINV1.157bp, pINV1.207bp and pEF1α, whereas INV1‐sp produced more conidia per lesion than WT (Mann–Whitney *U*‐test (each strain vs. WT): ***p < 0.001). Box plots show median, 25/75 percentiles and 1.5 × interquartile range. Background colours indicate growth rate and efficiency compared to the WT. See Table S3 for full data analysis and Figure S4 for pairwise comparisons.

To initiate infection, *M. oryzae* forms a specialised infection structure, an appressorium, to breach the host epidermis (Martin‐Urdiroz et al., 2016), which is crucial for successful foliar infection. Thus, we verified that all strains could effectively generate appressoria (Figure S5). We next conducted infection studies of rice plants with our strain collection during single‐strain infection. We found that the strains with different growth profiles on sucrose media *in vitro* also had different disease properties (Figure 4; Figure S4). Increasing growth rate compared to the wild‐type (Figure 3a: Strain pEF1α) led to decreased virulence, measured as area of the symptomatic necrotic disease lesion formed (Figure 4a). It also led to a decrease in the number of conidia produced per lesion (Figure 4b), a measure assumed to correlate with transmissibility (Sacristan & Garcia‐Arenal, 2008). Additionally, decreasing growth rate compared to the wild‐type altered disease properties non‐monotonically. Reducing growth rate to a small extent but without a resulting change in efficiency (Figure 3: Strain pINV1.207bp) led to reduced virulence compared to the wild‐type (Figure 4, Figure S4b). However, reducing growth rate further (as is the case with INV1‐sp), which then coincided with increased metabolic efficiency (Figure 3), increased virulence compared to the wild‐type (Figure 4; Figure S4e). In contrast, reducing growth rate further (Figure 3a: Strain pINV1.157bp), which also further enhances efficiency (Figure 3b), decreased virulence compared to the wild‐type. Therefore, we found that there is an optimum growth rate, constrained by the rate‐efficiency trade‐off, that maximises pathogen virulence and transmissibility during single strain infection.

### Intraspecific competition dictates growth‐strategy success

**FIGURE 5:**
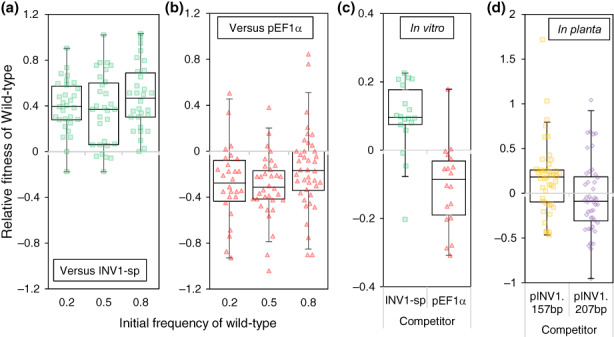
Competition experiments between strains with differing growth properties. Rice seedlings (CO‐39) were co‐inoculated with the wild‐type (WT‐Guy11) and either INV1‐sp (low rate, high efficiency) (a) or pEF1α (high rate, low efficiency) (b) using a leaf spot inoculation method. Initial frequency of WT was either 0.2, 0.5 or 0.8. WT had a higher fitness than INV1‐sp, but a lower fitness than pEF1α across all initial frequencies (one‐sample two‐sided Wilcoxon signed rank test, *μ* = 0: *p* < 0.001 for each initial frequency – full data analysis shown in Table S5). (c) *In vitro* pairwise competitions on 1% sucrose MM. WT was fitter than INV1‐sp (one‐sample two‐sided Wilcoxon signed rank test, *μ* = 0: *p* < 1.58 × 10^−3^, *V* = 154) but less fit than pEF1α (*p* < 1.05 × 10^−3^, *V* = 15). (d) WT had a higher fitness than the much slower growing strain, pINV1.157bp (one‐sample two‐sided Wilcoxon signed rank test, *μ* = 0, *V* = 725, *p* < 7.40 × 10^−3^, *n* = 44) but fitness was not significantly different against pINV1.207bp (*μ* = 0, *V* = 409, *p* = 0.318, *n* = 44) during *in planta* pairwise competitions initiated at a frequency of 0.5. Box plots show median, 25/75 percentiles and (the smaller of) 1.5 × IQR or min/max. Markers show all replicates with added horizontal noise to aid visualisation.

We subsequently explored the consequences for virulence evolution in diverse pathogen populations, frequently found in both natural and clinical settings (Balmer & Tanner, 2011; López‐Villavicencio et al., 2007; Tollenaere et al., 2012). To determine the relative fitness of different growth rate and efficiency characteristics, we tested whether faster but less efficient strains or slower but more efficient strains had a selective advantage during co‐infection. Rice was inoculated with a mixture of the wild‐type and one of the following strains: either a faster growing strain with reduced efficiency that had lower virulence when infecting alone (pEF1α), or a slower growing strain with increased efficiency that had higher virulence when infecting alone (INV1‐sp). Inoculations were initiated at a range of frequencies within a shared and localised infection site. Strains were tagged with a selectively neutral GFP marker to distinguish them during co‐infection (Figure S6). In both pairwise co‐infections, the faster growing strain with a lower efficiency had a selective advantage over the slower growing strain with higher efficiency (Figure 5a,b). To test whether this outcome resulted from the strains' metabolic properties, in the absence of host defences, we conducted equivalent co‐inoculations *in vitro* on agar‐supplemented sucrose media. In agreement with the *in planta* competitions, faster growing strains outcompeted slower growing strains (Figure 5c). Small‐scale *in planta* competitions were also conducted with the wild‐type against pINV1.157bp or pINV1.207bp at equal initial frequencies. The wild‐type had a selective advantage over the much slower growing strain pINV1.157bp, whereas neither strain had a significant advantage during wild‐type and pINV1.207bp co‐infection (Figure 5d). The latter is consistent with both competitors having similar growth properties (Figure 3a,b). Collectively, these *in planta* competition outcomes whereby faster growers outcompete slower growers indicate that growth rates measured *in vitro* (Figure 3a, Figure S3) are representative of those *in planta*.

## DISCUSSION

To investigate the influence of the RETO on the relationship between growth rate and virulence, we modified INV1 activity in the plant pathogen *M. oryzae* (Figure 2d). The strains generated had varied *in vitro* growth rates (Figures 2d and 3a), which were also indicative of within‐host multiplication rates, as demonstrated by co‐infection experiments where faster growing strains outcompeted slower growers (Figure 5). Increasing growth rate, however, coincided with reduced metabolic efficiency (Figure 3d) due to the RETO.

Our study is wide reaching. There is considerable evidence for the RETO in microbial metabolism, which constrains growth of many microbial species (Beardmore et al., 2011; Lipson, 2015; Nev et al., 2021; Pfeiffer et al., 2001; Postma et al., 1989). Moreover, pathogens frequently encounter environments where the RETO is at play, for example environments with resource heterogeneities that promote efficient metabolism (MacLean & Gudelj, 2006; Pfeiffer et al., 2001).

We found that the relationship between growth rate and virulence was non‐monotonic. From an initially low growth rate, increasing growth rate increased disease virulence, but only to a certain point, after which virulence decreased as growth rate increased further. This was because the RETO meant high growth rates were constrained by reduced efficiency. These findings support a novel hypothesis that, in addition to pathogen growth rate, growth efficiency also influences virulence, with the overall outcome determined by how the two properties interact (Lindsay et al., 2021). We suggest that the relationship between growth rate and virulence can be both positive and negative, depending on: (a) the growth rate‐metabolic efficiency interaction, and (b) how growth rate influences other disease processes, for example, the time‐dependent costs of overcoming host defences. In particular, rather than virulence simply being proportional to pathogen growth rate, as classically assumed (Anderson & May, 1982; Bremermann & Pickering, 1983; Choisy & de Roode, 2010; Frank, 1996; Lenski & May, 1994; Levin & Bull, 1994; Nowak & May, 1994; van Baalen & Sabelis, 1995), we propose a new relationship where virulence is jointly shaped by the interaction between growth rate and metabolic efficiency (see (2) in Supplementary Information). We now provide an intuitive explanation for the proposed relationship, applicable to a wide range of host–pathogen systems.

First, we discuss why slow‐growing pathogens can be more virulent than fast growers. Despite nutrient availability being limited within the host (Foster et al., 2016), we reason that increased pathogen load permits access to additional nutrient reserves when they proliferate into previously uncolonised host tissue. Thus, it can be envisaged that since slow but efficient growth increases growth yield under nutrient limitation, such pathogens could spread into previously unaccessed host tissue, making them more virulent than fast growers. Indeed, our outcomes are consistent with this intuition when comparing the growth rate, efficiency and virulence between the wild‐type strain Guy11, and the engineered strains INV1‐sp and pEF1α. In particular, INV1‐sp has a lower growth rate but higher efficiency than Guy11, resulting in a higher virulence. Similarly, pEF1α has higher growth rate but lower efficiency than Guy11, resulting in a lower virulence (Figures 2a,b and 3a).

Next, we discuss why given the above link between virulence and metabolic efficiency, an increase in efficiency will not always lead to increased virulence. For example, pINV1.157bp has higher metabolic efficiency (and lower growth rate) than INV1‐sp, yet it has lower virulence. This is consistent with virulence being a function of growth rate multiplied by efficiency (see (2) in Supplementary Information), so that efficiency × growth rate of pINV1.157bp can be a lower value than efficiency × growth rate of INV1‐sp. It could also be explained by factors that influence virulence, other than a pathogen's resource consumption. In addition to the ability to metabolise sucrose from the host (Lindsay et al., 2016), successful *M. oryzae* infection relies on the metabolically costly production of a battery of secreted effector proteins to suppress plant immunity or detoxify plant counterattacks. For example, hosts mount a defence response to resist infecting pathogens, such as by the production of reactive oxygen species (ROS) to reduce pathogen growth (Tanabe et al., 2009). In turn, pathogens expend energy to counteract host defences, such as by producing effector proteins that suppress plant immunity (Fernandez & Orth, 2018; Martin‐Urdiroz et al., 2016), or producing glutathione peroxidase to detoxify ROS (Huang et al., 2011). Since pathogens can experience a trade‐off between allocating resources towards growth or virulence factor production (Cui et al., 2019; Peyraud et al., 2016; Sturm et al., 2011), we argue that the production costs depend on growth rate (see (7) in Supplementary Information). This is because slow growers are more sensitive to stress than fast growers as they are relatively more starved and so less able to fuel costly gene expression to overcome host defences (Peyraud et al., 2016). Therefore, we postulate that the cost of resisting host defences is higher for pINV1.157bp than for INV1‐sp. Consequently, this increased cost could counteract the efficiency advantage of pINV1.157bp (Figure 3b), making it less virulent than INV1‐sp (Figure 4a).

To test whether ROS inhibits pINV1.157bp more than Guy11 because of its slower nutrient acquisition rate, we repeated the metabolic efficiency tests where pINV1.157bp had an advantage over Guy11 (Figure 3b), but this time in the presence of ROS (1 mM H_2_O_2_). We found pINV1.157bp lost its metabolic efficiency advantage (Figure S7). Moreover, when strains differ in growth rates but not efficiencies, like pINV1.207bp and Guy11, the faster grower is expected to have lower cost of resisting host defences and be more virulent, as found (Figures 3a,b and 4a).

Although the proposed relationship between pathogen growth and virulence takes a pathogen perspective, the biological basis of the [cost] term could also be considered from the host perspective. In particular, if virulence is a consequence of host resource consumption, then virulence could be lessened by hosts replenishing the consumed resources, such as by photosynthesising more sucrose. This replacement of consumed resources would be expected to be more effective when infected by pathogens that consume resources slowly. Other aspects of the RETO could also influence microbial growth and virulence that are not examined here. For instance the biochemical basis of the RETO is varied, but rapid metabolism can accumulate metabolic intermediates, such as ethanol, or cause an imbalance between anabolism and catabolism (MacLean & Gudelj, 2006; Beardmore et al., 2011).

To the best of our knowledge, no previous study makes a direct connection between the RETO and the growth rate‐virulence relationship. While some studies infer pathogen growth rates from endpoint pathogen load measures (e.g. De Roode et al., 2008; Pagán et al., 2007), these measures could, instead, be indicative of growth yield where the RETO is present since they only quantify growth at one timepoint. Our proposed growth rate‐efficiency–virulence relationship is also compatible with studies that find a positive growth rate–virulence relationship because there the RETO may be weak or non‐existent. This may occur if the metabolic pathways were operating sufficiently far from the thermodynamic trade‐off limit (Novak et al., 2006), which could happen when microbes do not experience nutrient limitations that primarily select for rapid growth (Pfeiffer et al., 2001).

In addition to the RETO, the growth rate–virulence relationship can also be confounded by disease‐system‐specific intricacies such as allocating energetic resources to promoting successful infection rather than growth (Cui et al., 2019; Meyer et al., 2010; Peyraud et al., 2016; Sturm et al., 2011), represented in our phenomenological model as a reduction in pathogen growth associated with counteracting host defences (see (7) in Supplementary Information). As such, pathogen growth may not always be the primary driver of virulence evolution (Chapuis et al., 2011; Tardy et al., 2019). Instead, differences in other traits, including the ability to manipulate host immunity (Frank & Schmid‐Hempel, 2008), virulence factor repertoires and immune detection may account for virulence variations. We were unable to entirely remove the possibility that growth rate interacts with secondary pathogen traits that influence virulence. However, our synthetic collection of pathogen strains minimised such complications, enabling us to focus on the influence of growth properties on virulence. The range of mutants available in *M. oryzae* (Eseola et al., 2021) affecting effector function, invasive growth and immune suppression will, however, enable a similarly controlled and systematic exploration of such factors in future. As rice is *M. oryzae*'s natural host, the metabolism, growth phenotype and infection processes of the wild‐type fungus have evolved in this environment. This provides resilience to our study since we avoid potential problems associated with model laboratory hosts that could confound experimental outcomes. Such problems include novel pathogen–host interactions and non‐natural within‐host conditions to which the pathogen may be maladapted. Although *M. oryzae* metabolises various host‐derived carbon sources (Foster et al., 2016), unsuccessful infection of mutants unable to metabolise sucrose suggests that any influence of the RETO while consuming them is relatively weak (Lindsay et al., 2016).

Our findings have profound consequences for understanding virulence evolution where selection acts on pathogens to increase their growth rate or efficiency. During co‐infection and when competition is mediated by resources, faster growing and more virulent strains are predicted to displace slower growing, less virulent strains and drive the evolution of increasing virulence (Frank, 1996; Nowak & May, 1994). In agreement, our study finds that faster growing pathogens are favoured during co‐infection (Figure 5). However, instead of necessarily increasing virulence, as traditionally assumed (Anderson & May, 1982; Bremermann & Pickering, 1983; Choisy & de Roode, 2010; Frank, 1996; Levin & Bull, 1994; Nowak & May, 1994; van Baalen & Sabelis, 1995), faster growing pathogens can be less virulent than slower growing pathogens when their metabolism is constrained by the RETO (Figure 4). Reduced efficiency means that faster growers generate fewer conidia (Figure 4b), which can be reasonably assumed to lower transmission (Sacristan & Garcia‐Arenal, 2008). We therefore demonstrate how, in contrast to the virulence‐transmission trade‐off, resource competition can drive a reduction in disease virulence despite low transmission. Thus, the RETO could be one of numerous factors contributing towards the breakdown of the virulence‐transmission trade‐off hypothesis (Acevedo et al., 2019; Alizon et al., 2009).

Our proposed relationship between growth rate, efficiency and virulence, suggests that the evolutionary stable strategy could lead to an intermediate level of virulence by balancing metabolic efficiency, which can enhance pathogen virulence and transmissibility when infecting alone (Figure 4), with rapid growth that promotes relative fitness during co‐infection (Figure 5). On the one hand, the RETO enables slower growers to have enhanced efficiency, providing them with a selective advantage under favourable conditions, such as a spatially structured environment (Lindsay et al., 2018; Lion & Boots, 2010; Pfeiffer et al., 2001). On the other hand, increasing growth rate can enhance pathogen fitness during co‐infection via exploitative competition (Figure 5). Thus, we hypothesise that a pathogen's optimal growth properties will be influenced by the frequency of co‐infections, which can be high for plant pathogens (López‐Villavicencio et al., 2007; Tollenaere et al., 2012). The degree of spatial segregation between pathogens is expected to fluctuate during disease outbreaks, which could enable diverse metabolic strategies to persist (Meyer et al., 2015).

The RETO may also explain why the wild‐type strain is less virulent than the engineered strain, INV1‐sp, during single genotype infections (Figure 4). Unlike our controlled single‐strain infections, in nature *M. oryzae* is likely to experience resource competition from co‐infecting strains within diverse populations found in the field (Saleh et al., 2014), or interspecific competition from later‐colonising saprophytes (Cui et al., 2019). Both cases may promote the evolution of rapid resource consumption and growth, thus limiting the efficiency and virulence of the wild‐type compared to INV1‐sp (Figures 3 and 4). The wild‐type might also be less efficient and virulent than INV1‐sp due to selection pressures from modern agricultural practices, such as monocultures of host plants (Yang et al., 2019). This selection may favour rapid growth because high conidia yields were unnecessary for transmission in monocultures where transmission efficiency is high.

Microbial growth rate and efficiency are key determinants of how microbial communities and ecosystems function, but their interaction has previously been overlooked in the virulence evolution literature (Beardmore et al., 2011). The RETO has already been acknowledged to act during diverse processes including the evolution of cooperation (Lindsay et al., 2018), community productivity (MacLean et al., 2010), the maintenance of biodiversity (Meyer et al., 2015) and ecological processes such as nutrient cycling (Malik et al., 2019). Incorporating it into various aspects of virulence evolution theory may further the understanding of epidemiological dynamics and how virulence changes over the course of epidemics. This includes understanding how vaccination, transmission modes and host spatial structuring influence selection on virulence (Cressler et al., 2016). This knowledge may be crucial for effectively predicting and managing disease.

## METHODS

### Strains, growth conditions and DNA analysis

*M. oryzae* strains are derived from the wild‐type Guy11 (Leung et al., 1990). Standard practices were used for maintenance, growth, DNA extraction and transformation (Talbot et al., 1993), and nucleic acid assessment and manipulation (Sambrook et al., 1989).

*In vitro* growth measurements were conducted on minimal media (MM) (Talbot et al., 1997) with D‐sucrose or D‐glucose at the specified concentrations and 15 g L^−1^ agar for solid media. Inoculum was conidia from strains cultured for 10–12 days on complete media (CM) (Talbot et al., 1993). Conidia were collected in H_2_O and filtered through Miracloth (Merck Millipore) to remove mycelial debris.

The *in vitro* growth properties of *M. oryzae* strains with altered *INV1* expression were assessed on sucrose MM at 26°C with 12 h/12 h light/dark cycles. Growth rate was measured as biomass formation per time in liquid media and growth efficiency was measured as conidiation per resource on agar‐supplemented media (See Supplementary Methods for more details).

### Generating *M. oryzae* strains with altered sucrose metabolism

Sucrose metabolism was modified by altering *INV1* (MGG_05785) expression, which encodes invertase (Lindsay et al., 2016) (β‐d‐fructofuranoside fructohydrolase, EC 3.2.1.26, glucose hydrolase family 32 [GH32]), by manipulation of the *INV1* promoter or deletion of the secretion signal peptide (Figure 2c) (see Supplementary Methods for more details).

### Enzymatic assay of invertase

Invertase activity was measured on live mycelium by determining the concentration of reducing sugars formed from sucrose hydrolysis using a colorimetric assay (see Supplementary Methods for more details).

### In planta studies

Rice plant (*Oryzae sativa* cultivar CO 39 – indica) infections were conducted using a quantitative and localised leaf spot inoculation method, as described previously (Lindsay et al., 2016). Virulence was measured using image analysis (ImageJ, National Institutes of Health, USA) to quantify the area of the symptomatic disease lesions after 7 days. *In planta* pathogen fitness was quantified by conidia production after lesions were placed under high humidity for 3 days (see Supplementary Methods for more details).

#### AUTHOR CONTRIBUTIONS

Ivana Gudelj, Richard J. Lindsay and Nicholas J. Talbot conceived the idea, designed experiments and wrote the manuscript, Richard J. Lindsay and Philippa J. Holder conducted the experiments, Ivana Gudelj and Richard J. Lindsay analysed the data, Ivana Gudelj developed the mathematical model and performed numerical simulations.

## Supporting information


Data S1.

Data S2.

## Data Availability

All data and code are available at https://doi.org/10.6084/m9.figshare.15052347
