# Supplementary material for: Metabolic efficiency reshapes the seminal relationship between pathogen growth rate and virulence
Source: Ecol Lett. 2023 Apr 13;26(6):896–907. doi: 10.1111/ele.14218 (PMC10947253; doi:10.1111/ele.14218)
Supplement: Supplementary file 1 — Data S1. [file ELE-26-896-s001.pdf]

Supplementary Information 2 – Metabolic efficiency reshapes the seminal relationship between pathogen growth rate and virulence

Contents

Supplementary Methods 1

In vitro growth measurements 1

Generating *M. oryzae* strains with altered sucrose metabolism 3

Enzymatic assay of invertase 4

In planta studies 4

Data analysis 5

Redefining the relationship between growth rate and virulence 5

Supplementary Figure 10 8

References 9

Supplementary Methods

In vitro growth measurements

Growth rates were obtained from 9 ml liquid sucrose (1%) MM inoculated with 10^5 conidia in a 6-well suspension culture plate (Greiner Bio-one). Growth was quantified as dry weight biomass by destructively sampling populations at given time points. Biomass data is shown up to a point where biomass increases because once nutrients are depleted the biomass declines due to programmed cell death or senescence.

In vitro conidiation was enumerated from 25 ml agar cultures on 9 cm diameter Petri dishes that were inoculated with 10^5 conidia. Conidia were inoculated in H_2O in a 4x4 array (Supplementary Fig. 8a), allowed to dry and incubated for 12d, after which conidia were harvested and counted with a haemocytometer. A pilot experiment with the WT (Guy11) demonstrated that this was an appropriate timepoint to allow resource depletion (Supplementary Fig. 8b), which is important for accurately measuring metabolic efficiency (conidia per unit of resource). For competition experiments, strains were inoculated in separate patches of the array. The relative fitness of the wild-type (v) in

competition (both *in vitro* and *in planta*) were calculated according to changes in conidia frequency: $v = \frac{x_2(1-x_1)}{x_1(1-x_2)}$ where x_1 and x_2 are the initial and final frequency of the wild-type, respectively.

Thus, growth rate was measured as biomass formation in liquid media over time and growth efficiency was measured as conidiation per plate on agar-supplemented media. Importantly, these stages of the life cycle are most appropriate for investigating how growth influences the evolution of virulence in this pathogen. Growth within host tissue is primarily hyphal and is the life stage where maximising rate and resource capture helps individuals to out-compete coinfecting strains. On the other hand, conidiation is the most appropriate measure of metabolic efficiency because it is the life-stage where maximising yield maximize transmission to new hosts, which is often considered to be immediate for spore producing pathogens (Sacristan & Garcia-Arenal 2008).

Measuring these growth parameters was also necessary for the following reasons. Firstly, quantifying the fitness of filamentous fungi is challenging compared to unicellular microbes due to their multicellularity and complex life cycles (Pringle & Taylor 2002). For instance, measures of colony expansion may not be indicative of total growth since the growth rate of individual hyphae is localized at the tip, whereas population growth and biomass synthesis may occur throughout the colony (Prosser & Tough 1991) (Supplementary Fig. 9). The appropriate method of growth measurement must consider the underlying causation of expected differences. Although growth differences caused by mutations in morphological regulatory genes may only be detected by apical growth rate of hyphae and not biomass formation (Rhodes 2006), such measures were found to be inappropriate for our *INV1* mutants because the null mutant had similar hyphal extension rates, despite clear deficiencies in biomass formation (Supplementary Fig. 9). Therefore, in this study, population growth rate was measured as dry weight biomass over time in liquid culture.

Secondly, the impact of the RETO during invertase-mediated sucrose metabolism can be nullified in spatially unstructured environments where diffusion is rapid. In such conditions, high hexose concentrations that are generated in the vicinity of the cell surface from extracellular sucrose hydrolysis are quickly lost via diffusion, effectively reverting the conditions to a low resource environment where the RETO is weak (MacLean *et al.* 2010; Lindsay *et al.* 2016; Lindsay *et al.* 2018). Therefore, metabolic efficiency was measured as growth yield in terms of conidiation on solid media, which preserves localized hexose concentration spikes from sucrose hydrolysis (MacLean *et al.* 2010). Moreover, conidiation is intrinsically linked to metabolic efficiency because it is induced by resource depletion (Ricci *et al.* 1991; Kim & Lee, 2012). Indeed, we observed the strong inhibitory effect of an available carbon source on conidiation when culturing the wild-type strain on minimal media with high glucose concentrations, since other nutrients are expected to be exhausted before the carbon, hence conidiation is repressed (Supplementary Fig. 8c).

Note, it was also necessary to measure these different features of filamentous fungal growth in different ways for the following reasons. Firstly, liquid cultures are not conducive to

conidiation (Zhang *et al.* 2014). Secondly, conidia-based measurements of growth rate could also be problematic because conidiation is induced by nutrient limitation (Kim & Lee, 2012) and so is not a linear function of mycelial growth. Thirdly, quantifying biomass formation on solid media is practically challenging.

Generating *M. oryzae* strains with altered sucrose metabolism

DNA sequences were obtained from the Ensembl genome database (Kersey *et al.* 2017). PCR was performed with Phusion High-fidelity DNA polymerase (NEB) or CloneAmp HiFi PCR premix (Takara) for genetic modifications, or SapphireAmp Fast PCR Master Mix (Takara) for colony PCR. Plasmids were constructed by In-Fusion cloning (Clontech Laboratories). Primers used are shown in Supplementary Table 6.

Sucrose metabolism was modified by altering *INV1* (MGG_05785) expression, which encodes the enzyme invertase (Lindsay *et al.* 2016) (β-d-fructofuranoside fructohydrolase, EC 3.2.1.26, glucose hydrolase family 32 (GH32)), by manipulation of the *INV1* promoter or secretion signal peptide. The wild-type strain Guy11 was used as a baseline of *INV1* expression and growth properties, from which modifications generated an additional four strains. In each strain, *INV1* expression was modified using one of three approaches: promoter swapping, 5' promoter truncations, or secretion signal peptide deletion (Fig. 2c). *INV1* is constitutively expressed at low levels, upregulated in response to sucrose, and expressed throughout the rice infection process (Fig. 2a) (Lindsay *et al.* 2016; Jeon *et al.* 2020; Yan *et al.* 2022). Gene expression is regulated by its upstream promoter region, to which transcription factors recruit RNA polymerase. Hence, modifying the promoter region can alter transcription levels (Soanes *et al.* 2002). Firstly, an *INV1* overexpressing strain was generated by swapping the native *INV1* promoter (*pINV1*) with that of the elongation factor 1-alpha gene *EF1α* (MGG_03641), which has high constitutive expression, both *in vitro* and *in planta* (Jeon *et al.* 2020). Secondly, we modified *INV1* expression by generating two *M. oryzae* strains with different length 5' truncations of *pINV1* (Fig. 2c). *INV1* deletion causes a loss of sucrolytic activity (Lindsay *et al.* 2016) (Fig. 2b). Based on the catalysis of sucrose hydrolysis, strains with a truncated *pINV1* of length 157bp (pINV1.157bp) or 207bp (pINV1.207bp) upstream of the start codon had reduced *INV1* expression compared to the wild-type (Fig. 2d, Supplementary Fig. 1).

The third approach for modifying *INV1* activity was to delete its secretion signal-peptide (Fig. 2c). Newly synthesized proteins destined for the secretory pathway generally possess a secretion signal peptide sequence in the N-terminal portion of the amino-acid chain, which is cleaved off as the protein is translocated through the endoplasmic reticulum membrane (Rapoport 1992; Nielsen *et al.* 1997). Signal peptide deletion can result in the production of a functional, intracellular form of the protein (Kaiser & Botstein 1986), which can decrease growth rate and increase efficiency (Lindsay *et al.* 2019). Therefore, we generated a strain (INV1-sp) with an *INV1* signal peptide sequence deletion by allelic replacement with the ORF, lacking base pairs 4-57 (Supplementary Fig. 2).

Reduced extracellular invertase activity was detected in the resulting strain compared to the wild-type (Fig. 2d, Supplementary Fig. 1).

Constructs containing the *INV1* ORF, 3'UTR and differing promoters detailed above were generated in the previously developed Strataclone (Stratagene) plasmid that contains the *BAR* gene conferring resistance to glufosinate ammonium (150 $mg.ml^{-1}$) (Lindsay *et al.* 2016). Vectors were integrated into the genome of the invertase deletion strain (Δ*inv1*) (Lindsay *et al.* 2016).

The *INV1* signal peptide was identified using SIGNALP 4.1 software (Petersen *et al.* 2011), which predicted a cleavage site between amino acids 19 and 20. We deleted the secretion signal peptide by allelic replacement of the wild-type gene with a modified version lacking this sequence. A transformation vector was generated with the following features: homology to the left flank region of *INV1,* the *INV1* ORF omitting nucleotides 4-57 and 0.5 kb 3'UTR, the *ILV1* gene, and region of homology to the right flank. A positive transformant was verified by Sanger sequencing (Supplementary Fig. 2).

Strains were distinguished in mixed-genotype populations by expression of a cytoplasmic GFP (*SGFP*) by the *ToxA* promoter (Sesma & Osbourn 2004), as previously (Lindsay *et al.* 2016). Fluorophore expression did not impose a detected fitness cost (Two-sample two-sided t-test: $p = 0.455$, $t = 0.765$; Supplementary Fig. 6).

Enzymatic assay of invertase

M. oryzae mycelium was grown in CM for 48 h which was then washed and transferred into sucrose MM for 4 h to induce expression. Live mycelium (approximately 50 mg dry weight) was then washed and transferred to 5 ml 1% sucrose in 0.1 M sodium acetate, pH 4.5, incubated at 30 ºC for 30 min. Invertase activity was determined based on the concentrations of reducing sugars formed from sucrose hydrolysis, detected by combining 100 µl of supernatant with 2.9 ml 0.5% (w/v) 4-Hydroxybenzhydrazide in 0.5 M NaOH, as described previously (Lindsay *et al.* 2016).

***In planta* studies**

Rice plant (*Oryzae sativa* cultivar CO 39 – indica) infections were conducted using a quantitative and localised leaf spot inoculation method, as described previously (Lindsay *et al.* 2016). Briefly, 20 µl droplets of 5 x 10^4 $conidia.ml^{-1}$ suspensions were inoculated onto attached rice leaves from 3-4-week-old rice plants (3 leaf stage) and kept under saturating humidity for 48 h to facilitate appressorium formation. The ability of all strains to form appressoria was tested by inoculating conidial suspensions onto hydrophobic coverslips and quantifying successful formation by microscopy after 24 h. Successful formation was considered to be > 75% (Parker *et al.* 2008). Infections proceeded for 7 d under normal rice growth conditions (12 h/12 h: dark/light - 25/27°C – 90/80 % RH). This inoculum level is representative of disease epidemic conditions (Wilson & Talbot 2009). Disease virulence was quantified as the area of the foliar necrotic lesion formed, as frequently used in plant pathogen studies e.g., (Zhan *et al.* 2002; Lindsay *et al.* 2016; Stewart *et al.* 2016;

Young *et al.* 2018). Images of the symptomatic lesions were captured after 7 d using an Epson Expression 168 Pro scanner (1200 d.p.i.). Lesion areas were quantified using image analysis software (ImageJ, National Institutes of Health, USA). To quantify *in planta* pathogen conidia production, at the 7 d point disease lesions were excised from infected leaves and placed under high humidity to induce sporulation for 3 d. Conidiation was extracted by flooding the excised disease lesion with 100 µl H_2O and agitating the lesion surface to detach conidia, which were enumerated with a haemocytometer.

Data analysis

Statistical tests were performed using R v. 3.5.3 and Excel 2016. Pairwise comparisons were conducted with a two-sample two-sided Student's t-test (or Welch's t-test when unequal variances were found by F-test), or a two-sample two-sided Mann-Whitney test or a one-sample Wilcoxon rank sum test ($\mu = 0$) when data was not approximately normal. Comparisons of relative invertase expression (Fig. 2d, Supplementary Table 1), growth rate and metabolic efficiency (Fig. 3a-c, Supplementary Table 2) were made using One-way ANOVA, with between-strain comparisons made of the β coefficients of the linear model. Homogeneity of variance was tested by Fligner-Killeen test and standardised residuals were assessed by Shapiro-Wilk normality test to verify the assumptions of the parametric tests. Between-strain analysis of appressorium formation was assessed by Kruskal-Wallis rank sum test.

Redefining the relationship between growth rate and virulence

Let V denote pathogen virulence, here measured as the lesion size, and let r denote the pathogen growth rate. By combining data from Figures 2a and 3a we plot r against V (Supplementary Figure 10, black dots) and consider the classical assumption (Anderson & May 1982; Bremermann & Pickering 1983; Lenski & May 1994; Levin & Bull 1994; Nowak & May 1994; van Baalen & Sabelis 1995; Frank 1996; Choisy & de Roode 2010) that

$$V(r) = f(r) \qquad (1)$$

where f denotes **an increasing** function of r.

We argue for a need to re-consider this classical assumption and instead postulate that the relationship between growth rate and virulence should also take into account the efficiency of resource use denoted here by e. For simplicity, we re-define (1) as

$$V(r) = f(r \times e) \qquad (2)$$

but note that one could incorporate more complex functions of e into (1). We then use our synthetic system to illustrate that (2) provides a better fit to the data than (1) considering both a linear and non-linear forms of an increasing function f (Supplementary Figure 10, grey and blue lines).

In particular, we first consider the simplest case where f is an increasing linear function, $f(r) = a \times r + b$ with a and b denoting some positive constants. In that case the classical relationship (1) takes the form

$$V(r) = a \times r + b, \qquad (3)$$

while our newly proposed relationship (2) becomes

$$V(r) = a \times r \times e + b. \qquad (4)$$

We find that (4) provides better fit to data than (3) (Supplementary Figure 10a).

Next we consider a case where f is an increasing non-linear function, $f(r) = \frac{a \times r}{b+r}$ with a and b representing some positive constants. In that case the classical relationship takes the form

$$V(r) = \frac{a \times r}{b+r}, \qquad (5)$$

while our proposed relationship (2) becomes

$$V(r) = \frac{a \times r \times e}{b+(r \times e)}. \qquad (6)$$

Again we find that (6) provides a better fit to data than (5) (Supplementary Figure 10b).

Our examples illustrate that a simple inclusion of the efficiency of resource use alongside growth rate into the formulation of virulence, significantly improves fit to data compared to the classical assumption that virulence is solely an increasing function of growth rate (Anderson & May 1982; Bremermann & Pickering 1983; Lenski & May 1994; Levin & Bull 1994; Nowak & May 1994; van Baalen & Sabelis 1995; Frank 1996; Choisy & de Roode 2010).

To further improve the fit to data, our newly proposed relationship in (2) could be expanded to include additional impacts on growth denoted by $c(r)$ that result from host-pathogen interactions:

$$V(r) = c(r) \times f(r \times e). \qquad (7)$$

Although a development of a detailed mechanistic relationship between V, r and e is beyond the scope of this work, we briefly discuss motivations behind the forms $c(r)$ could take. For example, $0 < c(r) < 1$ could be an increasing function of r (see Supplementary Figure 10c for an example). This could be justified by assuming that: (i) slower growing pathogens acquire energy from the host at a lower rate than faster growing pathogens (Otterstedt *et al.* 2004), which is supported by enzymatic (Fig. 2a) and growth assays (Fig. 3b) conducted in this study; (ii) hosts mount a counterattack to infection, such as by producing reactive oxygen species (Tanabe *et al.* 2009); (iii) pathogens expend a fixed amount of energy per unit of time into nullifying the host's counterattack. In the latter case, slower growing pathogens allocate a higher proportion of their acquired nutrients to fuel resistance to host attack compared to fast growers, thus incurring a higher cost which would be reflected in a low $c(r)$. An equivalent cost could also arise from the production of virulence factors that manipulate host physiology to enable successful infection (Parker *et al.* 2009). We find that

adding $c(r)$ into our newly proposed definition of virulence in (2), as shown in (7), improves the fit to data (see a comparison of Supplementary Figure 10c with 10a, grey lines). Importantly, incorporating $c(r)$ into (2) outperforms a scenario where $c(r)$ is incorporated into the classical relationship (1) (Supplementary Figure 10c, grey vs blue line). This result is consistent with efficiency e playing a key role in shaping virulence alongside growth rate.

One could also consider c to be a non-monotonic function of the growth rate. In animal models of infection, host immune responses can be delayed when infected by slow growing pathogens meaning that they are cleared less effectively than faster growing pathogens. However, if pathogens grow sufficiently fast then they can “outrun” and exhaust the immune response (Davenport *et al.* 2009). This change in growth between fast and slow pathogens could lead to a non-monotonic relationship between growth rate and strength of immune response.

Supplementary Figure 10

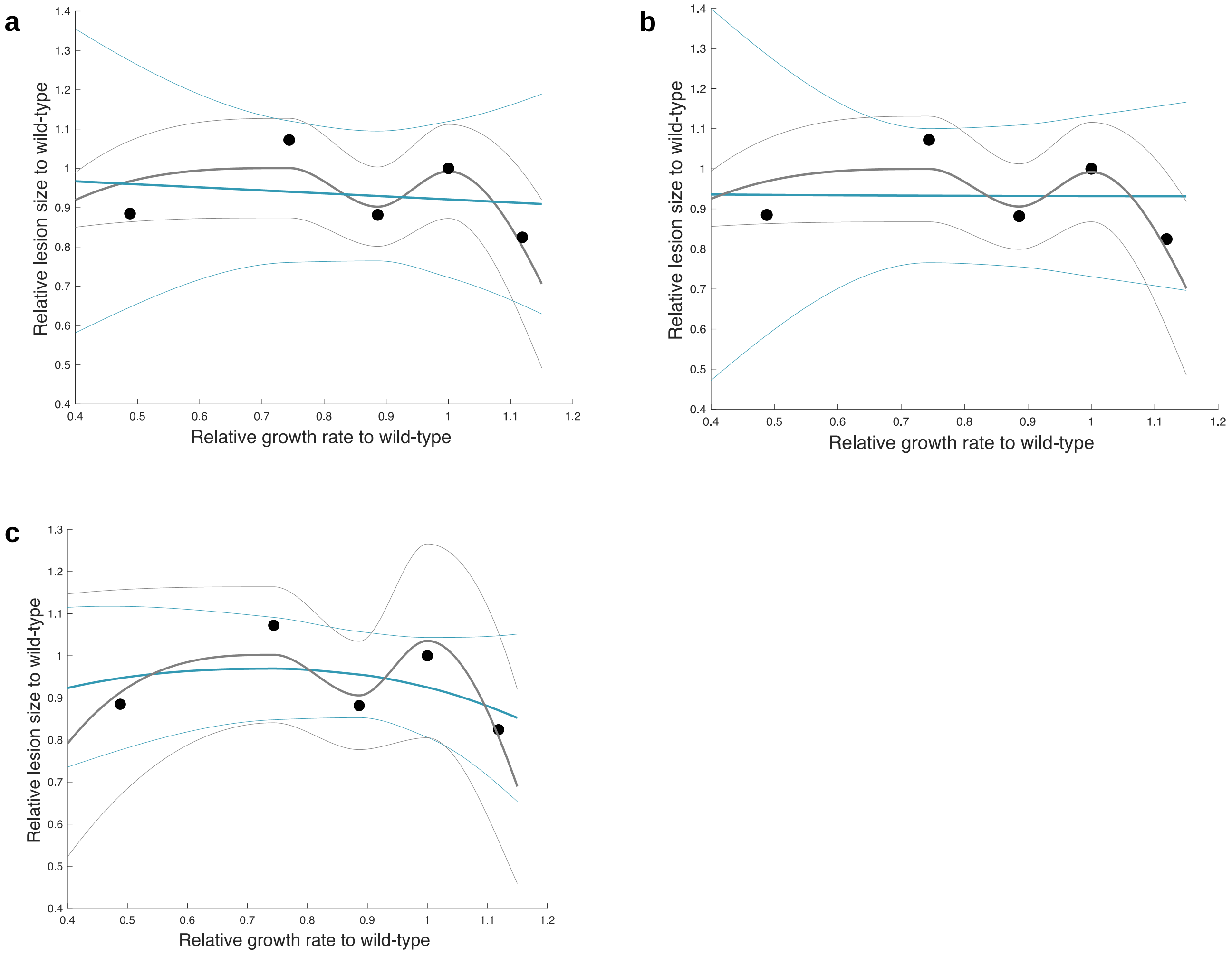

Supplementary Figure 10: The fitted relationship between growth rate and virulence comparing the classical virulence-growth relationship (blue) with the proposed inclusion of the efficiency of resource use (grey). Thicker lines represent the nonlinear regression fit to experimental data (means - black circles) while thin lines represent 95% confidence interval. (**a**) $V(r)$ in (3) denoted in blue and $V(r)$ in (4) denoted in gray were fitted where a and b are free parameters. (4) provides a better fit to data (AIC = -13.5914 and *adj* R^2 = 0.57) compared to (3) (AIC = -8.0537 and *adj* R^2 =-0.29). (**b**) $V(r)$ in (5) denoted in blue and $V(r)$ in (6) denoted in gray were fitted where a and b are free parameter. (6) provides a better fit to data (AIC = -13.3 and *adj* R^2 = 0.55) compared to (5) (AIC = -7.88 and *adj* R^2 = -0.33). (**c**) $V(r)$ from (7) denoted in grey was fitted to data with $f(r \times e) = a \times r \times e + b$ and $c(r) = \frac{1}{1+d \times \exp{(-r)}}$, where a, b, d are free parameters: AIC = -15.14 and *adj* R^2 = 0.62; In contrast, removing efficiency from (7), $V(r) = c(r)f(r)$ denoted in blue was fitted to data with $f(r) = a \times r + b$ and $c(r) = \frac{1}{1+d \times \exp{(-r)}}$, where a, b, d are free parameters yielding AIC = -7.8 and *adj* R^2 = 0.1.

References

Anderson, R.M. & May, R.M. (1982). Coevolution of hosts and parasites. *Parasitology*, 85 (Pt 2), 411-426.

Bremermann, H.J. & Pickering, J. (1983). A game-theoretical model of parasite virulence. *Journal of Theoretical Biology*, 100, 411-426.

Choisy, M. & de Roode, J.C. (2010). Mixed infections and the evolution of virulence: effects of resource competition, parasite plasticity, and impaired host immunity. *The American Naturalist*, 175, E105-118.

Davenport, M.P., Belz, G.T. & Ribeiro, R.M. (2009). The race between infection and immunity: how do pathogens set the pace? *Trends in Immunology*, 30, 61-66.

Frank, S.A. (1996). Models of parasite virulence. *Quarterly Review of Biology*, 71, 37-78.

Jeon, J., Lee, G.-W., Kim, K.-T., Park, S.-Y., Kim, S., Kwon, S. *et al.* (2020). Transcriptome profiling of the rice blast fungus Magnaporthe oryzae and its host *Oryza sativa* during infection. *Molecular Plant-Microbe Interactions*, 33, 141-144.

Kaiser, C.A. & Botstein, D. (1986). Secretion-defective mutations in the signal sequence for Saccharomyces cerevisiae invertase. *Molecular and Cellular Biology*, 6, 2382-2391.

Kersey, P.J., Allen, J.E., Allot, A., Barba, M., Boddu, S., Bolt, B.J. *et al.* (2017). Ensembl Genomes 2018: an integrated omics infrastructure for non-vertebrate species. *Nucleic Acids Research*, 46, D802-D808.

Kim, K.S. & Lee, Y.-H. (2012). Gene expression profiling during conidiation in the rice blast pathogen *Magnaporthe oryzae*.

Lenski, R.E. & May, R.M. (1994). The evolution of virulence in parasites and pathogens: reconciliation between two competing hypotheses. *Journal of Theoretical Biology*, 169, 253-265.

Levin, B.R. & Bull, J.J. (1994). Short-sighted evolution and the virulence of pathogenic microorganisms. *Trends in Microbiology*, 2, 76-81.

Lindsay, R.J., Kershaw, M.J., Pawlowska, B.J., Talbot, N.J. & Gudelj, I. (2016). Harbouring public good mutants within a pathogen population can increase both fitness and virulence. *Elife*, 5, e18678.

Lindsay, R.J., Pawlowska, B.J. & Gudelj, I. (2018). When increasing population density can promote the evolution of metabolic cooperation. *The ISME Journal*, 12, 849-859.

MacLean, R.C., Fuentes-Hernandez, A., Greig, D., Hurst, L.D. & Gudelj, I. (2010). A mixture of "cheats" and "co-operators" can enable maximal group benefit. *PLoS Biology*, 8, e1000486.

Nielsen, H., Engelbrecht, J., Brunak, S. & von Heijne, G. (1997). Identification of prokaryotic and eukaryotic signal peptides and prediction of their cleavage sites. *Protein Engineering, Design and Selection*, 10, 1-6.

Nowak, M.A. & May, R.M. (1994). Superinfection and the evolution of parasite virulence. *Proceedings of the Royal Society B*, 255, 81-89.

Otterstedt, K., Larsson, C., Bill, R.M., Ståhlberg, A., Boles, E., Hohmann, S. *et al.* (2004). Switching the mode of metabolism in the yeast Saccharomyces cerevisiae. *EMBO Reports*, 5, 532-537.

Parker, D., Beckmann, M., Enot, D.P., Overy, D.P., Rios, Z.C., Gilbert, M. *et al.* (2008). Rice blast infection of *Brachypodium distachyon* as a model system to study dynamic host/pathogen interactions. *Nature Protocols*, 3, 435-445.

Parker, D., Beckmann, M., Zubair, H., Enot, D.P., Caracuel-Rios, Z., Overy, D.P. *et al.* (2009). Metabolomic analysis reveals a common pattern of metabolic re-programming during invasion of three host plant species by *Magnaporthe grisea*. *The Plant Journal*, 59, 723-737.

Petersen, T.N., Brunak, S., von Heijne, G. & Nielsen, H. (2011). SignalP 4.0: discriminating signal peptides from transmembrane regions. *Nature Methods*, 8, 785-786.

Pringle, A. & Taylor, J. (2002). The fitness of filamentous fungi. *Trends in Microbiology*, 10, 474-481.

Prosser, J.I. & Tough, A. (1991). Growth mechanisms and growth kinetics of filamentous microorganisms. *Critical Reviews in Biotechnology*, 10, 253-274.

Rapoport, T.A. (1992). Transport of proteins across the endoplasmic reticulum membrane. *Science*, 258, 931-936.

Rhodes, J.C. (2006). *Aspergillus fumigatus*: growth and virulence. *Medical Mycology*, 44, S77-S81.

Ricci, M., Krappmann, D. & Russo, V. (1991). Nitrogen and carbon starvation regulate conidia and protoperithecia formation of *Neurospora crassa* grown on solid media. *Fungal Genetics Reports*, 38. 87-88.

Sacristan, S. & Garcia-Arenal, F. (2008). The evolution of virulence and pathogenicity in plant pathogen populations. *Molecular Plant Pathology*, 9, 369-384.

Sesma, A. & Osbourn, A.E. (2004). The rice leaf blast pathogen undergoes developmental processes typical of root-infecting fungi. *Nature*, 431, 582-586.

Soanes, D.M., Kershaw, M.J., Cooley, R.N. & Talbot, N.J. (2002). Regulation of the MPG1 hydrophobin gene in the rice blast fungus *Magnaporthe grisea*. *Molecular Plant-Microbe Interactions*, 15, 1253-1267.

Stewart, E.L., Hagerty, C.H., Mikaberidze, A., Mundt, C.C., Zhong, Z. & McDonald, B.A. (2016). An improved method for measuring quantitative resistance to the wheat pathogen *Zymoseptoria tritici* using high-throughput automated image analysis. *Phytopathology*, 106, 782-788.

Tanabe, S., Nishizawa, Y. & Minami, E. (2009). Effects of catalase on the accumulation of H_2O_2 in rice cells inoculated with rice blast fungus, *Magnaporthe oryzae*. *Physiologia Plantarum*, 137, 148-154.

van Baalen, M. & Sabelis, M.W. (1995). The dynamics of multiple infection and the evolution of virulence. *The American Naturalist*, 146, 881-910.

Wilson, R.A. & Talbot, N.J. (2009). Under pressure: investigating the biology of plant infection by *Magnaporthe oryzae*. *Nature Reviews Microbiology*, 7, 185-195.

Yan, X., Tang, B., Ryder, L., Maclean, D., Were, V.M., Eseola, A.B. *et al.* (2023). The transcriptional landscape of plant infection by the rice blast fungus *Magnaporthe oryzae* reveals distinct families of temporally co-regulated and structurally conserved effectors. *Plant Cell, In press.*

Young, G., Cooke, L., Watson, S., Kirk, W., Perez, F. & Deahl, K. (2018). The role of aggressiveness and competition in the selection of *Phytophthora infestans* populations. *Plant Pathology*, 67, 1539-1551.

Zhan, J., Mundt, C.C., Hoffer, M. & McDonald, B.A. (2002). Local adaptation and effect of host genotype on the rate of pathogen evolution: an experimental test in a plant pathosystem. *Journal of Evolutionary Biology*, 15, 634-647.

Zhang, H., Wu, Z., Wang, C., Li, Y. & Xu, J.R. (2014). Germination and infectivity of microconidia in the rice blast fungus *Magnaporthe oryzae*. *Nature Communications*, 5, 4518.
