# Supplementary material for: Metabolic efficiency reshapes the seminal relationship between pathogen growth rate and virulence
Source: Ecol Lett. 2023 Apr 13;26(6):896–907. doi: 10.1111/ele.14218 (PMC10947253; doi:10.1111/ele.14218)
Supplement: Supplementary file 2 — Data S2. [file ELE-26-896-s002.pdf]

Supplementary Information 1 - Metabolic efficiency reshapes the seminal relationship between pathogen growth rate and virulence

Richard J Lindsay[1], Philippa J Holder[1], Nicholas J Talbot[2] and Ivana Gudelj[1]*

[1]Biosciences and Living Systems Institute, University of Exeter, Exeter, EX4 4QD, UK

[2]The Sainsbury Laboratory, University of East Anglia, Norwich Research Park, Norwich, UK

*Corresponding author: I.Gudelj@exeter.ac.uk

RJL: R.J.Lindsay@exeter.ac.uk, PJH: P.J.Holder@exeter.ac.uk, NJT: Nick.Talbot@tsl.ac.uk

Contents:

Supplementary Figures 1-9

Supplementary Tables 1-6

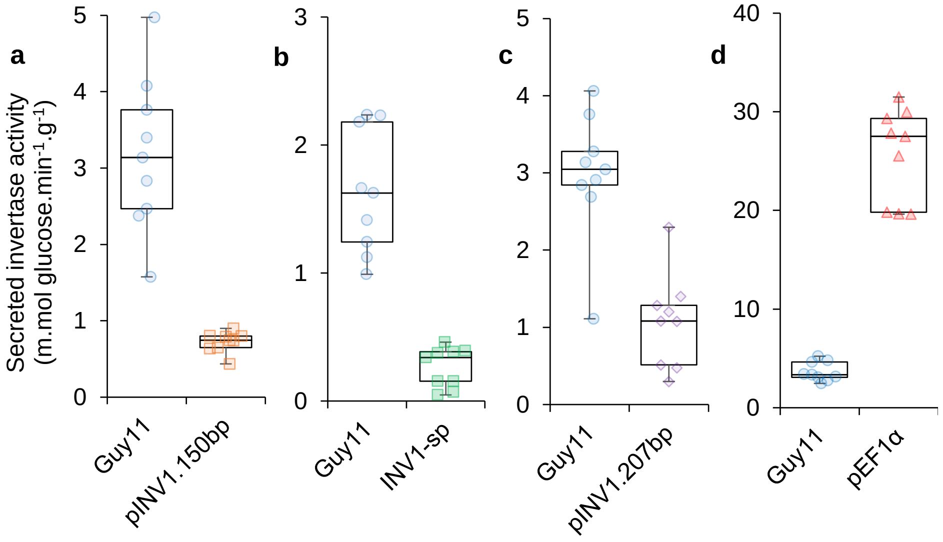

Supplementary Figure 1. Modifying *INV1* expression by 5' UTR modification – Pairwise test against the ancestral wild-type strain Guy11*.* Secreted invertase activity of the engineered strains were assessed by a colorimetric enzyme assay concurrently with the wild-type, Guy11. Expression was induced in 1% sucrose MM and tested in 1% sucrose at pH 4.5, 30°C. pINV1.157bp (**a**) INV1-sp (**b**) and pINV1.207bp (**c**) had lower activity than Guy11, whereas pEF1α (**d**) had increased activity (Two-sample two-sided t-test: $p < 0.0001$). Box plots show median, 25/75 percentiles and min/max. Points show all replicates, n = 9 collated from three experimental repeats. N.B. Vertical axes have different scales. Data normalised against the mean WT activity for each experimental repeat is shown in Fig. 2d. Full data analysis is shown in Supplementary Table 1.

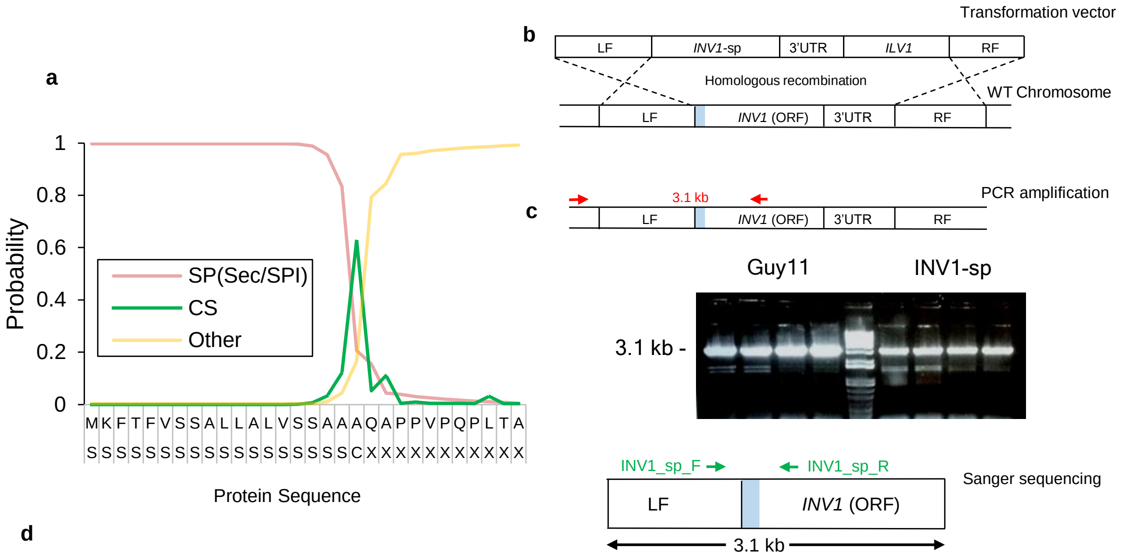

d

Sequencing results

Strain		Sequence																									
**70-15** MG8 Genome assembly	Nucleotide	ATG	AAA	TTC	ACA	TTT	GTG	TCA	TCG	GCG	CTT	CTG	GCG	CTC	GTC	TCC	TCC	GCC	GCC	GCT	CAA	GCC	CCT	CCG	GTG	CCC	---
	a.a	M	K	F	T	F	V	S	S	A	L	L	A	L	V	S	S	A	A	A	Q	A	P	P	V	P	-
**INV1-sp**	Nucleotide	ATG	---	---	---	---	---	---	---	---	---	---	---	---	---	---	---	---	---	---	CAA	GCC	CCT	CCG	GTG	CCC	---
	a.a	M	-	-	-	-	-	-	-	-	-	-	-	-	-	-	-	-	-	-	Q	A	P	P	V	P	-
**Guy11**	Nucleotide	ATG	AAA	TTC	ACA	TTT	GTG	TCA	TCG	GCG	CTT	CTG	GCG	CTC	GTC	TCC	TCC	GCC	GCC	GCT	CAA	GCC	CCT	CCG	GTG	CCC	---
	a.a	M	K	F	T	F	V	S	S	A	L	L	A	L	V	S	S	A	A	A	Q	A	P	P	V	P	-

Supplementary Figure 2: Generating a *M. oryzae* strain with secretion-deficient INV1. a SIGNALP 4.1 software predicted a signal peptide cleavage site between amino acids 19-20 (probability: 0.6284 – green dashed line), suggesting that the secretion signal peptide preceeded this (Secretion signal peptide sequence probability – pink line) and the sequence after does not have any kind of signal peptide (yellow-line). **b** To delete the *INV1* secretion signal peptide (blue shaded region) we generated a transformation vector for allelic replacement of the native locus with an *INV1* ORF that omits nucleotides 4-57. This vector had homology to the left flank (LF) region of *INV1,* the *INV1* ORF without the secretion signal peptide, 0.5 kb 3'UTR to include the terminator sequence, the *ILV1* gene conferring sulfonylurea (chlorimuron-ethyl) resistance, and a region of homology to the right flank (RF). LF and RF allow targeted in-frame transformation by homolgous recombination. **c** A positive transformant of the secretion signal peptide deletion (INV1-sp) was verified by Sanger sequencing. Firstly an amplicon was generated (3.1 kb) that extended upstream of the LF of the transformation vector (indicated by red arrows). The signal peptide sequence region was then sequenced forwards and backwards with the primers INV1_sp_F & R (Supplementary Table 6). **d** The sequencing results were aligned against the ancestral wild-type, Guy11, and the MG8 assembly of the *M. oryzae* (strain 70-15) genome, confirming the deletion of the desired region.

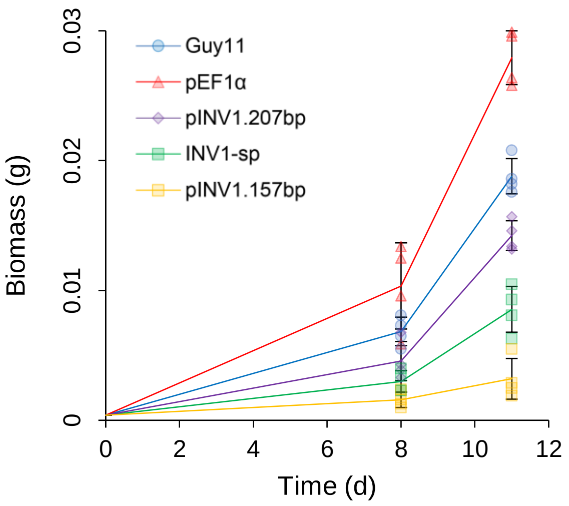

Supplementary Figure 3. Time series growth data of *M. oryzae* strain collection. Growth rates were measured by inoculating 10^5 conidia (≈ 400 μg d.w.) into 9 ml liquid 1% sucrose minimal media. Replicates were destructively harvested after 8 d or 11 d and dryweight (d.w.) biomass established. Values indicate growth rate (as opposed to yield) since they are taken prior to resources being exhausted, demonstrated by Guy11 establishing approximately 0.04 g d.w. in the same conditions after 16 d (Suplementary Fig. 9a). Malthusian growth parameters (m) were calculated for each data point where m = ln (final biomass/initial biomass) / d. It was found that d did not significantly influence on m (linear model: $p = 0.892$) so replicates from different values of d were collated (as shown in Fig. 3a) and assessed by linear model. Full pairwise analysis is shown in Supplementary Table 2. Line follows mean ± 95% C.I., n = 4.

Supplementary Figure 4 – 1/3

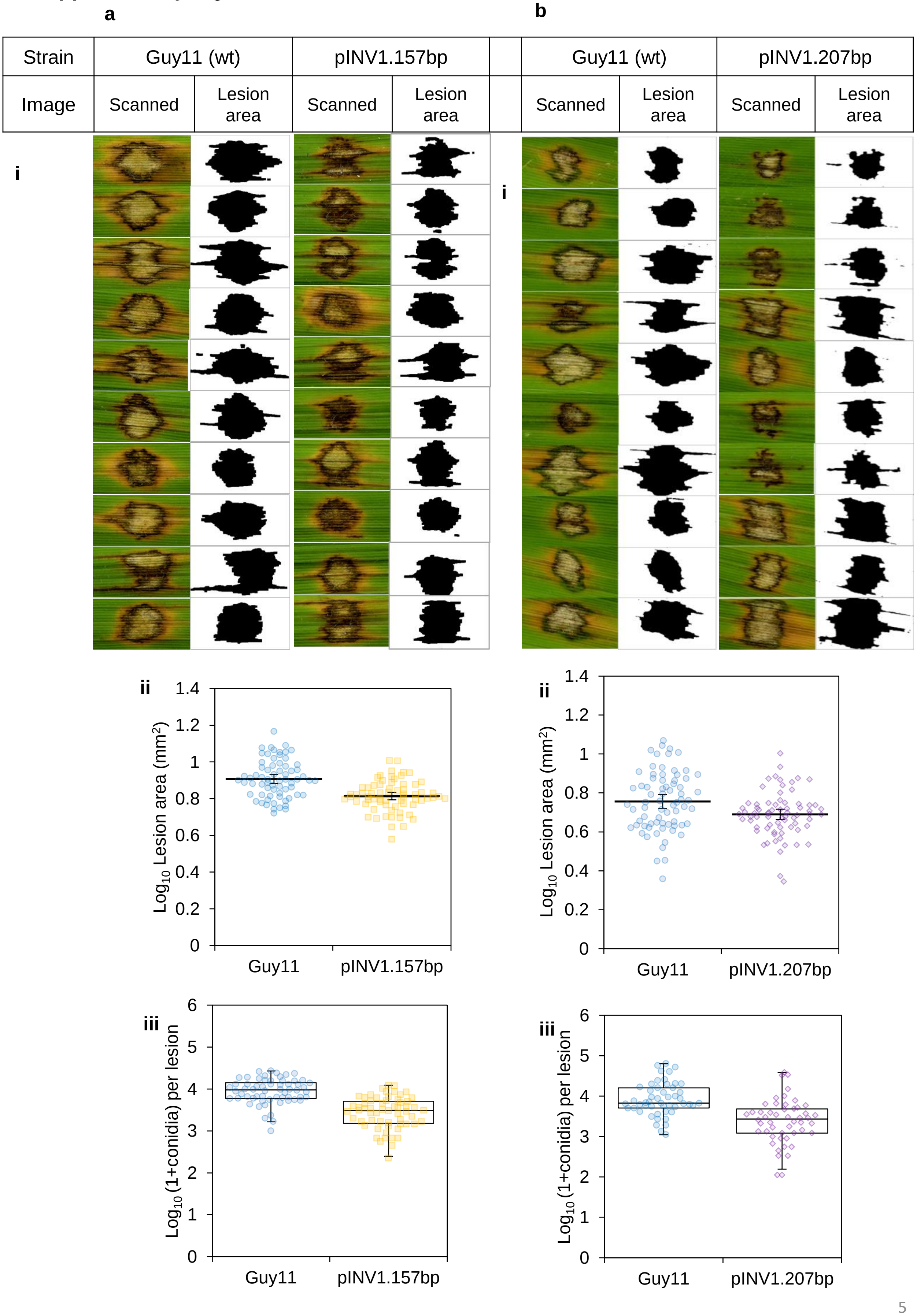

Supplementary Figure 4 – 2/3

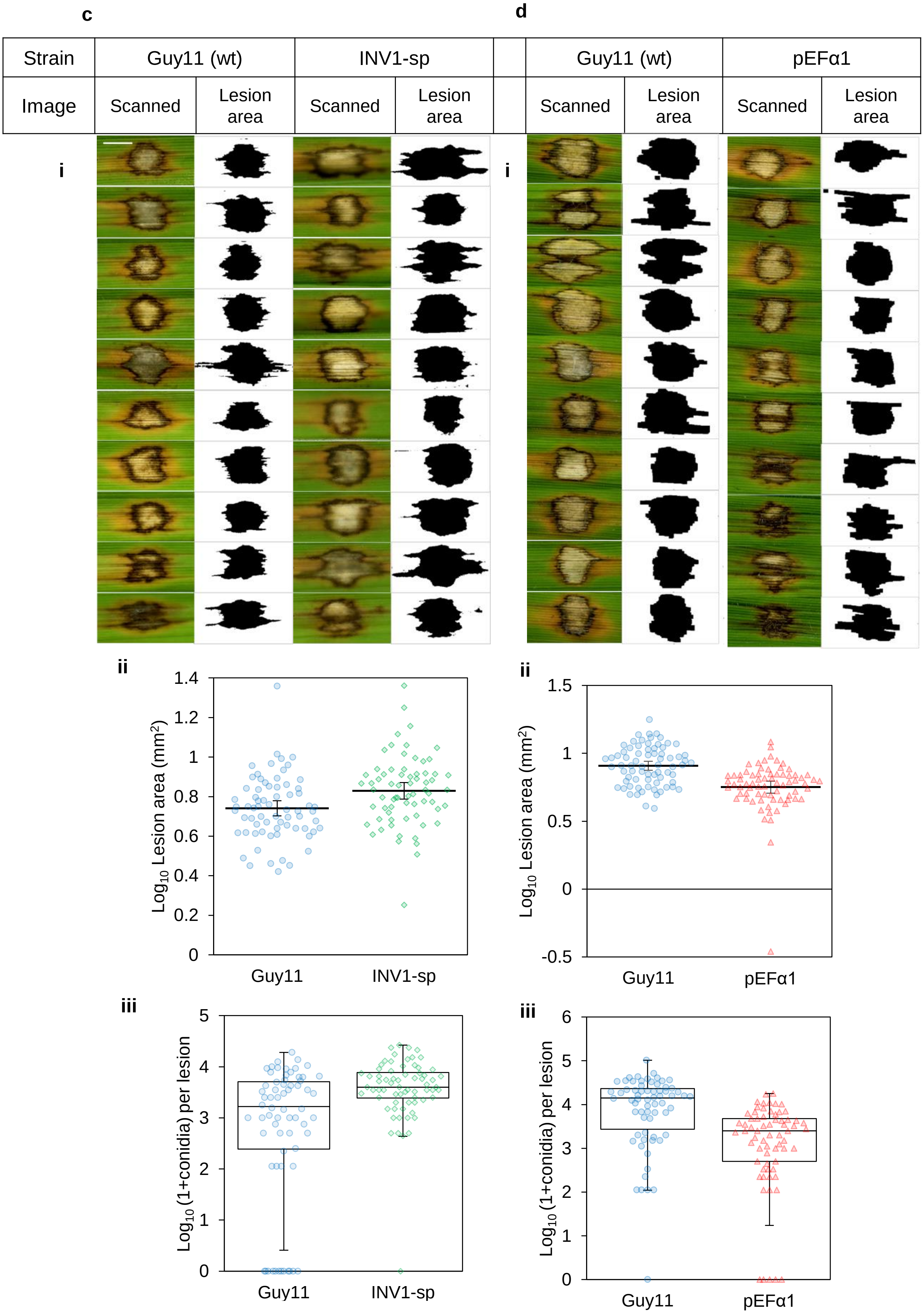

Supplementary Figure 4 - 3/3

Supplementary Figure 4: Infection assays to determine virulence and *in planta* pathogen multiplication. Rice seedlings (Cultivar Co-39 – 3-4 leaf stage) were infected by leaf drop inoculation with 10^3 conidia. Each mutant with altered growth properties was compared against the wild-type ancestral strain Guy11 (WT) (**a-d**). Disease virulence was quantified by imaging the sytomatic blast disease lesions after 7d and the lesion area measured by image analysis. For each strain combination, (**i**) shows image capture of 10 replicates (left, scale bar = 2 mm) and corresponding area that was measured (right), (**ii**) shows lesion area (mean ± 95% CI). After image capture, disease lesions were excised from leaves and placed under high humidity for 3d to induce conidiation, which was enumerated with a haemocytometer (**iii**). Box plots show median, 25/75 percentiles and 1.5 x interquartile range. WT was significantly more virulent ($p < 3.45 \times 10^{-3}$) and had higher conidiation ($p < 1.94 \times 10^{-7}$) than pINV1.157bp (**a**), pINV1.207bp (**b**) and pEF1α (**d**). INV1-sp was significantly more virulent ($p < 2.70 \times 10^{-3}$) and had higher conidiation ($p < 1.70 \times 10^{-4}$) than Guy11 (**c**). Data was normalised against the mean WT lesion area and the median conidiation for each combination for Figure 4. Data collated from at least three experimental repeats. Full data analysis is shown in Supplementary Table 3 and 4.

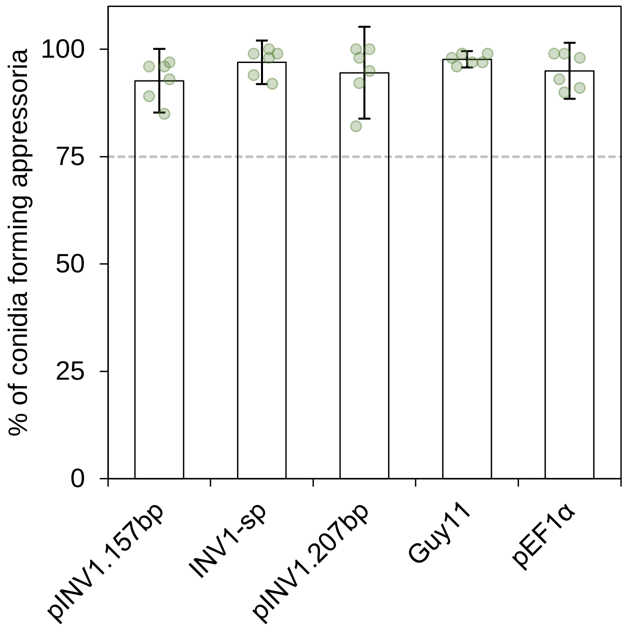

Supplementary Figure 5: *M. oryzae* forms an appressorium to breach the plant epidermis for hyphal invasion of plant tissue. All strains were tested for their ability to form appressorium on hydrophic coverslips after 24 h. All strains successfully generated appressorium at a rate of > 75% (viable frequency according to Parker *et al.,* 2008), shown by dashed line, and had no significant differences between strains (Kruskal-Wallis rank sum test: χ^2 = 1.338, p = 0.8549). Experiment was performed twice, with replicates collated. Mean ± 95% CI, n = 6 with at least 100 conidia assessed per replicate.

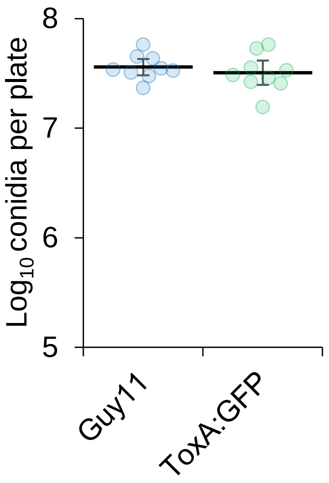

Supplementary Figure 6: Strains were tagged with a constitutively expressed cytoplasmic GFP from the *ToxA* promoter to distinguish them during co-inoculation. Guy11 and Guy11 tagged with the GFP (ToxA:GFP) were assessed for fitness costs by quantifying conidiation after 12d on 1% sucrose MM agar plates (25 ml). The GFP did not impose a significant selective cost (two-sample two-sided t-test: p = 0.455, t = 0.765). Experiment was performed in triplicate. Mean ± 95% CI, n = 9.

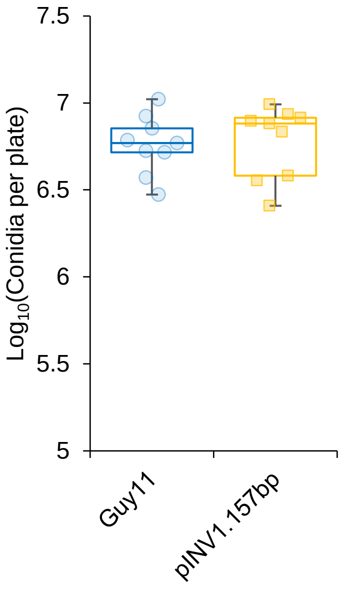

Supplementary Figure 7: It was postulated that the costs of resisting and recovering from host counterattack to infection depends on growth rate because slow growing pathogens are less able to fuel costly gene expression to overcome defences. One such enzyme that *M. oryzae* produces is glutathione peroxidase to detoxify ROS produced by the host [54]. To test the costs of resisting and recovering from host defences we measured conidiation of on 1% sucrose MM in the presence of ROS (1 mM H_2O_2). We inoculated the wild-type Guy11 and strain pINV1.157bp (slow growing, high efficiency) in the same manner as before where pINV1.157bp had an efficiency advantage over Guy11 in the absence of H_2O_2 (Fig. 3b). In the presence of H_2O_2, pINV1.157bp lost its efficiency advantage over the Guy11 (two-sample two-sided t-test: $t = 0.355$, $p = 0.727$, $n = 9$), thus providing evidence that slower growing strains are less able to fuel gene expression to overcome host defences.

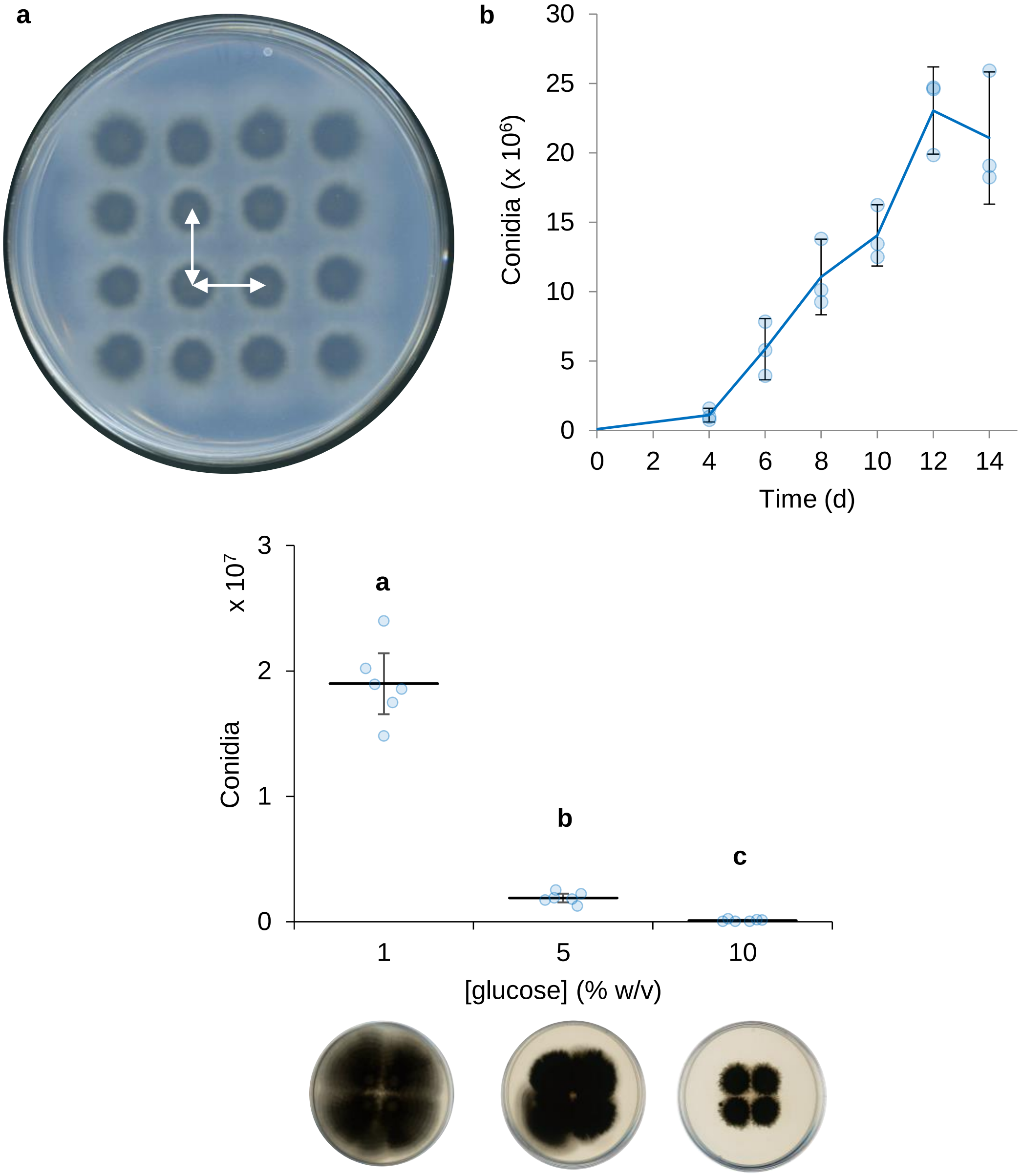

Supplementary Figure 8: *In vitro* conidiation assays were conducted on 9 cm diameter single vented Petri dishes on 25 ml MM (15 g/l agar) supplemented with D-sucrose or D-glucose at the specified concentrations. Plates were inoculated with a total of 10^5 conidia per plate. **a** Conidia were inoculated in a 4x4 array. The mid-point of each patch were separated by 15mm (white arrows). For competition experiments, strains were inoculated into separate patches of the 4x4 array to simulate the spatially structured environment that pathogens inhabit within the host. **b** A pilot experiment was conducted with Guy11 to assess the appropriate timepoint to harvest conidia when resources were depleted. Conidiation saturated by day 12, so this timepoint was used for subsequent conidiation assays. Line follows mean (± 95% C.I.), n = 3. **c** To test the inhibitory effect of carbon source availability on conidiation,10^5 conidia of the wild-type were inoculated onto the central 2x2 array of MM plates containing different glucose concentrations. We found that high glucose concentrations inhibited conidiation (linear model: adj. R^2= 0.9446, $F_{(2,15)}$= 145.8, $p < 1.482 \times 10^{-10}$), despite observable fungal growth (see inset images). Conidia were enumerated and images captured after 12d. Mean (± 95% C.I.), n = 6, different letters indicate significant differences between [glucose] (β coefficients: $p < 0.0001$ – response variable (conidia) was Log_{10}-transformed).

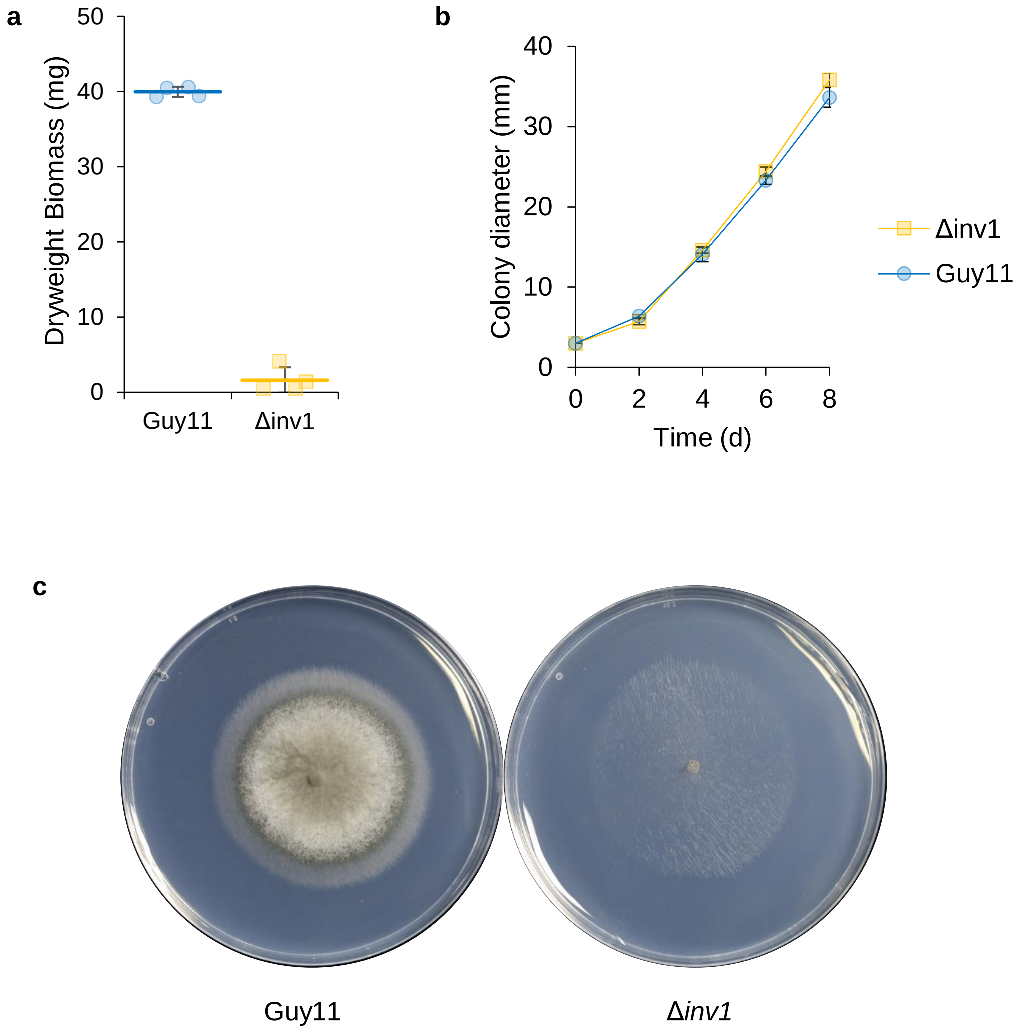

Supplementary Figure 9: Measuring growth rate.
The invertase deletion mutant Δ*inv1* does not effectively metabolise sucrose and cannot form substantial biomass when sucrose is the sole carbon source (biomass after 16 d, Welch's t-test: Δ*inv1* > 0: p = 0.152, t = 1.91; two-sample two-sided t-test: Guy11 > Δ*inv1*: $p < 1.29 \times 10^{-8}$, t = 41.7, n = 4, mean ± 95% CI, points show all replicates) (**a**). Nevertheless, it has a slightly higher colony expansion rate on agar-supplemented sucrose plates than the WT (Guy11) (**b**) (linear model of d2-d8 growth data (adj. $R^2 = 0.9918$, $F_{(3,35)} = 1525$, $p < 2.2 \times 10^{-16}$) time:strain interaction term $p < 1.68 \times 10^{-3}$, mean ± 95%CI, n = 5). This expansion is made up of fine hyphae rather than the dense, melanised hyphae that is typical of the WT when grown on 1% sucrose MM + 1.5% agar no. 2 (**c**). This growth might be driven by trace amounts of metabolizable carbon sources in the agar. Colony expansion is, therefore, an inappropriate measure of growth rate for our system.

Pairwise comparisons vs. wild-type

Figure	Strain 1	Strain 2	F-test for homogeneity of variance (p-value)	T-test (two-tailed)	T-stat	P-value
2a	Guy11	Δ*inv1*	2.22×10^{-8}	Welch's	16.34	1.98×10^{-7}
S1a	Guy11	pINV1.150bp	2.94×10^{-6}	Welch's	7.19	9.32×10^{-5}
S1b	Guy11	INV1-sp	2.12×10^{-3}	Welch's	8.03	1.14×10^{-5}
S1c	Guy11	pINV1.207bp	0.197	Student's	5.58	4.13×10^{-5}
S1d	Guy11	pEF1α	8.82×10^{-5}	Welch's	13.6	2.63×10^{-7}

Between-strain analysis of relative activity

Linear model: $F_{(3,32)} = 127.8$, $p < 2.2 \times 10^{-16}$. Adj $R^2 = 0.9158$.

Fligner-Killeen test of homogeneity of variances: $\chi^2 = 5.7186$, $p = 0.1261$.

Shapiro-Wilk normality test of standardised residuals: W = 0.96327, p = 0.2707.

Linear model between-strain comparisons:

(*β* coefficients p < 0.0001 ***, < 0.01 **, < 0.05 *, NS – non-significant > 0.05)

Strain 1	Strain 2	p	Sig.
pINV1.150bp	INV1-sp	0.0174	*
pINV1.207bp	INV1-sp	0.000584	**
pEF1α	INV1-sp	$< 2.2 \times 10^{-16}$	***
pINV1.207bp	pINV1.150bp	0.200	NS
pEF1α	pINV1.150bp	2.99×10^{-16}	***
pEF1α	pINV1.207bp	3.65×10^{-15}	***

N.B. Between-strain relative activity data was Log_{10} transformed to meet assumptions of parametric test.

Supplementary Table 1. Modifying *INV1* expression by 5' UTR modification – Data analysis. Pairwise comparisons of strains were assessed by two-sided, two-sample t-test (Welch's t-test for non-homogenous variance). Full pairwise dataset is shown in Supplementary Figure 1. Between-strain analysis of relative expression (data shown in Figure 2d) was assessed with a linear model (relative activity ~ strain) using p-values of *β* coefficients.

Between-strain growth rate analysis (plotted in Fig. 3a):

Linear model: $F_{(4,35)} = 61.29$, $p < 2.54 \times 10^{-15}$. Adj $R^2 = 0.861$.

Linear model between-strain comparisons (β coefficients p < 0.0001 ***, < 0.01 **, < 0.05 *)

Fligner-Killeen test of homogeneity of variances: $\chi^2 = 4.4214$, p = 0.352.
Shapiro-Wilk normality test of standardised residuals: W = 0.98378, p = 0.8253.

Strain 1	Strain 2	p	Sig.
pINV1 150bp	INV1-sp	1.40×10^{-6}	***
pINV1 207bp	INV1-sp	0.0027	**
pEF1α	INV1-sp	4.91×10^{-10}	***
pINV1 207bp	pINV1 150bp	1.13×10^{-10}	***
Guy11	pINV1 150bp	1.48×10^{-13}	***
pEF1α	pINV1 150bp	3.45×10^{-16}	***
Guy11	pINV1 207bp	0.0143	*
pEF1α	pINV1 207bp	7.02×10^{-6}	***
pEF1α	Guy11	0.0108	*
Guy11	INV1-sp	1.38×10^{-6}	***

Between-strain metabolic efficiency analysis (1% Sucrose plotted in Fig. 3b, 0.01% Sucrose in Fig. 3c):

Linear model (excluding Δinv1 †): **1%**: $F_{(4,40)} = 33.14$, $p < 3.28 \times 10^{-12}$, Adj. $R^2 = 0.745$.

Fligner-Killeen test of homogeneity of variances: $\chi^2 = 2.2242$, p = 0.6946.
Shapiro-Wilk normality test of standardised residuals: W = 0.95829, p = 0.1051.

0.01%: $F_{(4, 40)} = 0.6252$, p = 0.6472, Adj. $R^2 = -0.0353$.

Fligner-Killeen test of homogeneity of variances: $\chi^2 = 0.74651$, p = 0.9455.
Shapiro-Wilk normality test of standardised residuals: W = 0.98083, p = 0.6533.

Linear model between-strain comparisons

(β coefficients p < 0.0001 ***, < 0.01 **, NS – non-significant > 0.05):

Strain 1	Strain 2	1% Sucrose		0.01% Sucrose	
		p	Sig.	p	Sig.
pINV1.150bp	INV1-sp	1.48×10^{-5}	***	0.975	NS
pINV1.207bp	INV1-sp	0.00382	**	0.734	NS
pEF1α	INV1-sp	9.53×10^{-7}	***	0.332	NS
pINV1.207bp	pINV1.150bp	7.88×10^{-10}	***	0.758	NS
Guy11	pINV1.150bp	1.33×10^{-9}	***	0.240	NS
pEF1α	pINV1.150bp	2.55×10^{-13}	***	0.348	NS
Guy11	pINV1.207bp	0.86777	NS	0.383	NS
pEF1α	pINV1.207bp	0.00982	**	0.526	NS
pEF1α	Guy11	0.00637	**	0.809	NS
Guy11	INV1-sp	0.00597	**	0.228	NS

† Δinv1 was included in the experiments as a negative control but was not included in the statistical analysis since its inclusion violated the assumptions of the parametric test on 1% sucrose (Fligner-Killeen test: p = 0.03046)

RETO (Fig. 3d): Linear model (on mean values for each strain): $F_{(1,3)} = 81.89$, $p < 2.86 \times 10^{-3}$, Adj. $R^2 = 0.953$.

Shapiro-Wilk normality test of standardised residuals: W = 0.99589, p = 0.9957.

Supplementary Table 2. Between-strain growth rate and metabolic efficiency analysis. Between-strain comparisons were assessed using a linear model (growth rate: *m* ~ Strain, metabolic efficiency: condia yield ~ Strain) using p-values of β coefficients. Time-series of growth is shown in Supplementary Fig. 3 with extracted Malthusian growth parameters *(m)* shown in Fig. 3a.

Strain 1	Strain 2	Strain 1 (n =)	Strain 2 (n =)	Strain 1 Log_{10} mean	Strain 2 Log_{10} mean	F-test p-value	T-test (two-tailed)	T-stat	P-value
Guy11	p157 INV1	67	67	0.908	0.814	0.055	Student's	5.79	4.82×10^{-8}
Guy11	p207 INV1	72	72	0.756	0.689	1.18×10^{-2}	Welch's	2.98	3.45×10^{-3}
Guy11	pEF1α	72	72	0.909	0.751	2.74×10^{-4}	Welch's	5.58	1.32×10^{-7}
Guy11	INV1-sp	69	68	0.741	0.829	0.235	Student's	3.06	2.70×10^{-3}

Supplementary Table 3. Infection assays to determine virulence – Data analysis. Pairwise comparisons of strains' lesion area (log_{10} lesion area (mm^2)) were assessed by two-sided, two-sample t-test (Welch's t-test for non-homogenous variance (by F-test)). Full pairwise dataset is shown in Supplementary Figure 4. Normalised data to mean Guy11 lesion area is shown in Figure 4.

Strain 1	Strain 2	Strain 1 (n =)	Strain 2 (n =)	Strain 1 Log_{10}(**median**+1)	Strain 2 Log_{10}(**median**+1)	W	P-value
Guy11	pINV1.157bp	60	60	3.977619	3.493055	3115.5	5.10×10^{-12}
Guy11	pINV.207bp	50	50	3.827563	3.434268	2000.5	1.94×10^{-7}
Guy11	pEF1α	66	66	4.531492	3.398114	946.5	2.10×10^{-8}
Guy11	INV1-sp	68	68	3.221143	3.602169	1448.5	1.70×10^{-4}

Supplementary Table 4. Infection assays to determine *in planta* conidiation – Data analysis. Pairwise comparisons of strains' *in planta* conidiation (log_{10} 1 + conidia) were assessed by Mann-Whitney U-test. Full pairwise dataset is shown in Supplementary Figure 4. Normalised data to median Guy11 conidia is shown in Figure 4.

One-sample two-sided Wilcoxon signed rank test:

Competitor	Initial frequency of wild-type	Median relative fitness of Wt	p	V	n
INV1-sp	0.2	0.3972	3.89×10^{-6}	431.5	30
INV1-sp	0.5	0.3680	1.63×10^{-5}	442.5	30
INV1-sp	0.8	0.4688	2.69×10^{-6}	435	30
EF1α	0.2	-0.2774	1.01×10^{-4}	43	30
EF1α	0.5	-0.3128	2.99×10^{-5}	24	30
EF1α	0.8	-0.1673	2.19×10^{-3}	148	40

Supplementary Table 5. *In planta* competition experiments between strains with differing growth properties – data analysis from Fig. 5a-b. One-sample two-sided Wilcoxon signed rank tests were performed at each initial frequency for each strain combination against equal fitness ($\mu = 0$). Normal approximation of p-value.

Construct	Primer name	Nucleotide sequence (5’ - 3’)
**INV1 expression vectors**	pINV1_207_F_pSC	TGCAGCCCAATGTGGAATTCAGCATACGTACGGAGCAAAG
	pINV1_157_F_pSC	TGCAGCCCAATGTGGAATTCGAAGAGGAAAAAAAAAAAGG
	INV1_3UTR_R_pSC	TCGACGGTATCGATAAGCTTTATGTCGGCTGTCTTTCTCC
	pEFα1_F_pSC	TGCAGCCCAATGTGGAATTCGTATTGTTTGGTGACCCTTTGC
	pEFα1_R	TTTGGCGGTTTGGTGCTCTC
	INV1_F_pEFα1	GAGAGCACCAAACCGCCAAAATGAAATTCACATTTGTGTCATCGG
**Transformation vector for signal peptide deletion of INV1**	pINV1_F_pSC	ATCCACTGTGGAATTCCAGATTATGTGTATGGCGGC
	pINV1_R_IF	CATCGTGCTGGTATTATTAA
	INV1_ORF-SP_F	AATACCAGCACGATGCAAGCCCCTCCGGTGCCCCA
	INV1_3UTR_R	ATGTCGGCTGTCTTTCTCCA
	ILV_F_INV1	AAAGACAGCCGACATGTCGACGTGCCAACGCCACAGTGC
	ILV_R	GTCGACGTGAGAGCATGCAATTCC
	INV1_RF_F_ILV	TGCTCTCACGTCGACAGGGATGAATTTGTGATAATACCG
	INV1_RF_R_pSC	GCCCAATGTGGAATTCCATGTTAAAATAAACTCTTGCCAC
**INV1-sp mutant sequencing**	Seq_F	TTCATACCTGTGGAAGATCGCT
	Seq_R	GCGCCGAGAAACTTCCAATC
	INV1_sp_F	TGATCTCGGGTTCTCCGGCTTC
	INV1_sp_R	ACCGCAAGGGTCGTTCATCC

Supplementary Table 6: Primer sequences used in this study. Underlined regions indicate complimentary regions for In-fusion cloning or fusion PCR.
